# An Integrated Pharmacology-Based Strategy to Investigate the Potential Mechanism of Xiebai San in Treating Pediatric Pneumonia

**DOI:** 10.3389/fphar.2022.784729

**Published:** 2022-02-14

**Authors:** Zhuohui Luo, Jiawen Huang, Ennian Li, Xinqian He, Qiqi Meng, Xinan Huang, Xiaoling Shen, Changkai Yan

**Affiliations:** ^1^ Department of Pharmaceutics, School of Pharmaceutical Sciences, Peking University, Beijing, China; ^2^ Honz Pharmaceutical Co., Ltd., Haikou, China; ^3^ Science and Technology Innovation Center, Guangzhou University of Chinese Medicine, Guangzhou, China; ^4^ Guangdong Provincial Key Laboratory of Clinical Research on Traditional Chinese Medicine Syndrome, The Second Clinical Medical College, Guangzhou University of Chinese Medicine, Guangzhou, China; ^5^ Artemisinin Research Center, Guangzhou University of Chinese Medicine, Guangzhou, China

**Keywords:** network pharmacology, molecular docking, molecular dynamics simulation, experiment verification, Xiebai San, pediatric pneumonia

## Abstract

Xiebai San (XBS) is a traditional Chinese medicine (TCM) prescription that has been widely used to treat pediatric pneumonia since the Song dynasty. To reveal its underlying working mechanism, a network pharmacology approach was used to predict the active ingredients and potential targets of XBS in treating pediatric pneumonia. As a result, 120 active ingredients of XBS and 128 potential targets were screened out. Among them, quercetin, kaempferol, naringenin, licochalcone A and isorhamnetin showed to be the most potential ingredients, while AKT1, MAPK3, VEGFA, TP53, JUN, PTGS2, CASP3, MAPK8 and NF-κB p65 showed to be the most potential targets. IL-17 signaling pathway, TNF signaling pathway and PI3K-Akt signaling pathway, which are involved in anti-inflammation processes, immune responses and apoptosis, showed to be the most probable pathways regulated by XBS. UPLC-Q/Orbitrap HRMS analysis was then performed to explore the main components of XBS, and liquiritin, quercetin, kaempferol, licochalcone A and glycyrrhetinic acid were identified. Molecular docking analysis of the compounds to inflammation-associated targets revealed good binding abilities of quercetin, kaempferol, licochalcone A and liquiritin to NF-κB p65 and of quercetin and kaempferol to Akt1 or Caspase-3. Moreover, molecular dynamics (MD) simulation for binding of quercetin or kaempferol to NF-κB p65 revealed dynamic properties of high stability, high flexibility and lowbinding free energy. In the experiment with macrophages, XBS markedly suppressed the (Lipopolysaccharides) LPS-induced expression of NF-κB p65 and the production of pro-inflammatory cytokines IL-6 and IL-1β, supporting XBS to achieve an anti-inflammatory effect through regulating NF-κB p65. XBS also down-regulated the expression of p-Akt (Ser473)/Akt, Bax and Caspase-3 and up-regulated the expression of Bcl-2, indicating that regulating Akt1 and Caspase-3 to achieve anti-apoptotic effect is also the mechanism of XBS for treating pediatric pneumonia. Our study helped to reveal the pharmacodynamics material basis as well as the mechanism of XBS in treating pediatric pneumonia.

## Introduction

Pneumonia is a rather common childhood condition mainly caused by bacteria, viruses, or fungi infection leading to children fighting for breath as their lungs fill with pus and fluid (Quinton et al., 2018). Despite high vaccination coverage, pneumonia is still the leading cause of morbidity and mortality in children under the age of five (Gupta, 2012; Goodman et al., 2019), claiming the lives of approximately 700,000 to 900,000 children every year (Troeger et al., 2017; WHO World Health Organisation Geneva, 2018). *Streptococcus pneumoniae* is the most commonly identified organism that causes bacterial pneumonia in children under five (GBD 2016 Lower Respiratory Infections Collaborators, 2016). Antibiotic medications can treat most bacterial pneumonias, but do not work on viruses and fungal pneumonias. The early experience-based administration of antibiotics, including amoxycillin, procaine penicillin, cefpodoxime, azithromycin, and erythromycin, did improve childhood pneumonia (Kabra et al., 2006), but long-term use of antibiotics is prone to drug resistance and side effects. Thus, novel and safe treatment strategies for pediatric pneumonia are urgently required.

Traditional Chinese medicine (TCM) has been widely used to prevent and treat various diseases because of its multiple pharmacological activities and good safety profile. Xiebai San (XBS), a well-known ancient TCM prescription, was first recorded in “XiaoEr YaoZheng ZhiJue” compiled by Yi Qian of the Song Dynasty and has been used clinically to treat pediatric pneumonia for hundreds of years (Zhang et al., 2020). XBS is composed of three traditional herbs, *Mori Cortex* (the dried root bark of *Morus alba* L*.*), *Lycii Cortex* (the dried root bark of *Lycium chinense* Mill.), and *Licorice* (the dried root and rhizome of *Glycyrrhiza uralensis* Fisch.)*.* Chemical studies have shown that flavones, and organic acids are the major ingredients in XBS (Chen et al., 2019; Cheng et al., 2021; Zheng et al., 2021). Similar to other TCM formulae, XBS involves multiple components, targets and pathways to exert pharmacological effects, whereas its main bioactive ingredients and molecular mechanism in anti-pediatric pneumonia remain poorly understood.

In recent years, network pharmacology has emerged as a more comprehensive method to explain the interactions between drugs and biological systems (Hopkins, 2007). Network pharmacology is an interdisciplinary approach, newly developed in the systematic research of drugs based on artificial intelligence and big data. It is widely applied to explain the correlation mechanism between the active ingredients of TCM and diseases in biomolecular networks that provides new ideas for Chinese medicine research based on a complex system. Meanwhile, computational biology, such as molecular docking (Fan et al., 2019) or dynamics simulation (Nunes-Alves et al., 2020), plays an important role in computer-aided drug design and development and is one of the core disciplines and methods in modern life technology.

The aim of this study was to explore the potential mechanism of XBS against pediatric pneumonia. First, we used the network pharmacology approach to predict and construct a “drug-ingredient-target-disease” network for XBS. Then, a UPLC-Q/Orbitrap HRMS method was performed to identify the main compounds in XBS. On this basis, molecular docking and dynamics simulation were carried out to analyze the interaction between the identified active ingredients and the predicted targets. Finally, to verify the predicted anti-pediatric pneumonia mechanism, expression of the predicted key targets involved in inflammation and apoptosis pathways were investigated in cultured macrophages. Our results provided a scientific basis for the clinical use of XBS for treating pediatric pneumonia.

## Materials and Methods

### Reagents and Chemicals

Quercetin, kaempferol, isorhamnetin, glycyrrhetinic acid, liquiritin, and glycyrrhizic acid ammonium salt were obtained from the National Institutes for Food and Drug Control (Beijing, China). Naringenin and licochalcone A were offered from Nanjing Spring and Autumn Biotech (Nanjing, China). Lipopolysaccharides (LPS; *Escherichia coli* 055: B5) were purchased from Solarbio LIFE SCIENCES (Beijing, China). The purity of each reference standard was above 98%. Cell Counting Kit 8 (CCK-8) was obtained from Dojindo Laboratory (Kumamoto, Japan). Fetal bovine serum (FBS) and RPMI 1640 medium were products of Biological Industries (Kibbutz Beit Haemek, Israel). Antibodies against Akt, Phospho-Akt (Ser473), Bcl-2, Bax and NF-κB p65 were supplied by Cell Signaling Technology (Beverly, MA, United States). Antibody for active + pro Caspase-3 was purchased from ABclonal Technology Co., Ltd. (Wuhan, China). Actin β Polyclonal Antibody was purchased from Immunoway (Suzhou, China). The Annexin V fluorescein isothiocyanate (FITC)/Propidium Iodide (PI) apoptosis kit was provided by the Beyotime Institute of Biotechnology (Shanghai, China). All other chemicals were provided by Sigma-Aldrich Co. (Shanghai, China), unless otherwise indicated.

### Establishment of the XBS-Ingredient-Target Interaction

In order to obtain the active ingredients of each herb in XBS, we utilized the Traditional Chinese Medicine System Pharmacology Database (TCMSP) (https://tcmspw.com/tcmsp.php), which is a uniquesystems pharmacology platform of Chinese herbal medicines that captures the relationship between drugs, targets, and diseases (Ru et al., 2014). We selected screening criteria including an oral bioavailability (OB) threshold ≥30% and a drug-likeness (DL) threshold ≥0.18 based on the ADME-related properties (absorption, distribution, metabolism, and excretion) of the candidate ingredients derived from TCMSP to identify the bioactive ingredients of XBS for subsequent research. To clarify the relationship between the active ingredients of XBS and pneumonia-related targets, we used the TCMSP database to collect the targets of each bioactive ingredient, while using multiple databases, including OMIM (https://omim.org/) (Amberger et al., 2015), Genecards (https://www.genecards.org/) (Rebhan et al., 1997), Drugbank (https://www.drugbank.ca/) (Wishart et al., 2008), DisgeNET (https://www.disgenet.org/) (Piñero et al., 2017) and DigSee (http://210.107.182.61/geneSearch/) (Kim et al., 2013) to obtain pneumonia and pediatric pneumonia-related targets of *Homo sapiens*. Subsequently, all pneumonia-associated targets were confirmed using UniProtKB ID and the name of each protein was obtained in the UniProt database (https://www.uniprot.org/) (UniProt Consortium, 2019). A gene database of targets of XBS was established by comparing and analyzing the overlapping targets between the pneumonia and the active ingredient targets of XBS.

### Construction of a Protein–Protein Interaction Network

The overlapping targets of active ingredients and pneumonia were considered as hub genes and analyzed using online STRING database (https://string-db.org/) (Szklarczyk et al., 2017) to obtain the protein–protein interaction (PPI) based on the minimum required interaction scores. Moreover, the TSV format file was downloaded and imported into the Cytoscape software (version 3.6.0) (Shannon et al., 2003) to construct and visualize the PPI network.

### Gene Ontology Enrichment and Pathway Analysis

To better elucidate the potential molecular mechanisms of XBS, we utilized the Metascape database (http://metascape.org/) (Zhou et al., 2019), which is a web-based portal that combines functional enrichment, interactome analysis, gene annotation and membership search, such as Gene Ontology (GO) enrichment analysis (biological processes, molecular function, and cellular component) and Kyoto Encyclopedia of Genes and Genomes (KEGG) pathway analysis, aiming to find out the biological function and molecular pathways of the selected targets.

### UPLC-Q/Orbitrap HRMS Analysis

The test freeze-dried aqueous extract of XBS according to the prescription proportion of “XiaoEr YaoZheng ZhiJue” of the Song Dynasty medical monograph was prepared by Honz Pharmaceutical Co., Ltd. (Guangzhou, China). Briefly, the mixture of *Mori Cortex* (41.3 g), *Lycii Cortex* (41.3 g), and *Licorice* (4.13 g) was soaked in 8-fold mass of purified water for 30 min and then refluxed for 1 h. After being filtered with gauze, the residue was refluxed with a 6-fold mass of purified water for another 1 h and filtered with gauze. The two filtrates were combined and concentrated using a rotary evaporator (70°C, −0.09 mpa). Finally, the concentrate was lyophilized using a freeze dryer with a yield of 16.9%. The qualitative analysis of the bioactive ingredients screened from network pharmacology was performed using Accela Ultra Performance Liquid Chromatography (UPLC) system coupled with Q-Orbitrap HRMS/MS (Thermo Fisher Scientific, United States). Chromatographic separation was carried out by using a Waters Acquity UPLC^○^R BEN C18 column (2.1 mm × 100 mm, 1.7 μm) at a flow rate of 0.2 ml/min with water (A) and methanol (B). The gradient elution was as follows: 0–15 min, 45% B; 15–18 min, 45–80% B; 18–21 min, 80–90% B; 21–31 min, 90% B. An aliquot volume (5 μL) of the filtered samples was injected for analysis. The MS analysis was performed using a positive mode equipped with a heated electrospray ionization (HESI) source. The parameters of optimized MS were as follows: the spray voltage in positive mode was 5.0 kV; the sheath gas pressure was 40 abr; the auxiliary gas pressure was 5 abr; the ion transfer capillary tube temperature was 320°C; the ion transfer capillary tube pressure was 6.0 V; and the RF lens RF was 90.0 V. Samples were analyzed through the acquisition mode of full scan MS/DD MS^2^ with a range of 100–1,000 m/z at the first-order mass resolving power of 30,000 in MS/MS. In addition, quercetin, isorhamnetin, kaempferol, glycyrrhetinic acid, glycyrrhizic acid ammonium salt, naringenin, licochalcone A and liquiritin (purity ≥ 98%) of reference standards were also detected. All data were acquired and processed by Xcalibur 2.2 software (Thermo Fisher Scientific, FL, United States).

### Molecular Docking

The interaction modes of the predicted active ingredients with the selected pneumonia-related targets were analyzed by means of molecular docking studies (Wang et al., 2003). Two- and three-dimensional structural information of XBS active ingredients were obtained from the PubChem database (https://pubchem.ncbi.nlm.nih.gov/) (Wang et al., 2009), and all the crystal structures of the targets used for molecular docking were retrieved from the RCSB protein data bank (https://www.rcsb.org/) (Burley et al., 2019). PDB_IDs of these targets are shown in Supplementary Table S1. In addition, the interaction modes between the selected targets and their inhibitors were also investigated. Molecular modeling was carried out using the SYBYL-X 2.2.1 program package.

### Molecular Dynamics Simulation

After the docking studies were completed, the active ingredients with good binding ability to the protein were further evaluated for their stability in the binding pocket using GROMACS v.2019.4 software package to run 100 ns molecular dynamics (MD) of protein-compound conjugates according to the previously described method (Das et al., 2020). The amber99sb-ildnff force field and the TIP3P water model were performed at a constant temperature and pressure and a periodic boundary condition. The force field of the micro-molecule was generated using the acpype.py script in AmberTools. During MD simulation, the hydrogen bond involved was constrained using the LINCS algorithm with 2.0 fs integration time step, and then the Particle Mesh Ewald (PME) method was applied to calculate the electrostatic interactions with the cutoff value set to 1.2 nm. The non-bonding interaction cutoff was 10 Å and was updated every 10 steps. In addition, the V-rescale temperature coupling and Berendsen method were used to keep the temperature and pressure constant at 300 K and 1 bar, respectively, to perform 100 ps NVT and NPT equilibrium simulation. After the completion of 100 ns simulation, analyses of trajectories and binding free energy were performed using different inbuilt scripts of GROMACS (Kumari et al., 2014), and the Pymol 2.4 (DeLano, 2002) programs were used for visualization.

### Cell Culture

The RAW 264.7 cells were maintained in RPMI 1640 medium with 10% FBS and antibiotics (100 U/mL penicillin and 100 μg/ml streptomycin) at 37°C in a 5% CO_2_ environment. In all the experiments, cells were allowed to acclimate for 24 h before any treatments.

### Cell Viability Assay

To investigate the effect of XBS on cell viability, cells were seeded into 96-well plates (3 × 10^4^ cells/well) and treated with different concentrations of XBS and LPS for 24 h. Cells without treatment were set as the control. Subsequently, cell viability was evaluated by a CCK-8 assay following the manufacturer’s instructions. Meanwhile, to investigate whether the pretreatment with XBS affects the viability of LPS-treated cells, cells were treated with different concentrations (100, 200 or 400 μg/ml) of XBS for 1 h prior to exposure to LPS (0.5 μg/ml). Subsequently, cells were incubated for 24 h and cell viability was evaluated as described above.

### Interleukin-6 and Interleukin-1β Measurement

Cells were pretreated with various concentrations of XBS for 1 h prior to exposure to LPS. Cells without any treatment were set as the normal control. After 24 h of stimulation, IL-6 and IL-1β in the culture media secreted by cells were measured using the corresponding ELISA kits (CUSABIO, Wuhan, China).

### Apoptosis Assay

Cells were plated into 6-well plates and pretreated with various concentrations of XBS for 1 h prior to exposure to LPS. After stimulation, cells were washed twice with cold PBS and examined with FITC-labeled annexin V and propidium iodide staining using the Annexin V-FITC Apoptosis Detection Kit (Beyotime Biotechnology, Shanghai, China) by following the manufacturer’s instructions, and then the percentages of apoptotic cells and necrotic cells were analyzed using flow cytometry (Beckman Coulter, United States).

### Western Blot Analysis

Cells with different treatments were lysed using RIPA lysis buffer containing PMSF and phosphatase inhibitors. An enhanced bicinchoninic acid (BCA) protein assay kit (Beyotime, Shanghai, China) was used for the quantification of the total protein in the lysis, and then the samples were mixed with a loading buffer (5×), heated for 5 min at 95–100°C and stored at −20°C. To detect the expression of indicated proteins, equal amounts of proteins were loaded and separated on 8–15% SDS-PAGE gels, and electro-transferred to PVDF membranes (Millipore Co., Ltd. MA, United States) using the traditional wet transferring system by Mini-PROTEAN Tetra System (BIO-RAD, CA, United States). Membranes were sealed with 5% nonfat milk or 5% BSA, and then incubated overnight at 4°C with relative primary antibodies. Then, the blots were followed by incubation with horseradish peroxidase (HRP)-conjugated secondary antibodies. Protein bands were detected with an enhanced chemiluminescence (ECL) reagent kit (Millipore, Darmstadt, Germany) by Molecular Imager® (BIO-RAD, CA, United States). ImageJ software (NIH, Bethesda, MD, United States) was used for bands pattern analysis.

### Statistical Analysis

The values were expressed as the mean ± SD and analyzed using SPSS 22.0. One-way ANOVA followed by the LSD test was used to assess the statistical differences. *p* values of 0.05 or less were considered statistically significant.

## Results

### Screening of Active Ingredients for XBS

**FIGURE 1:**
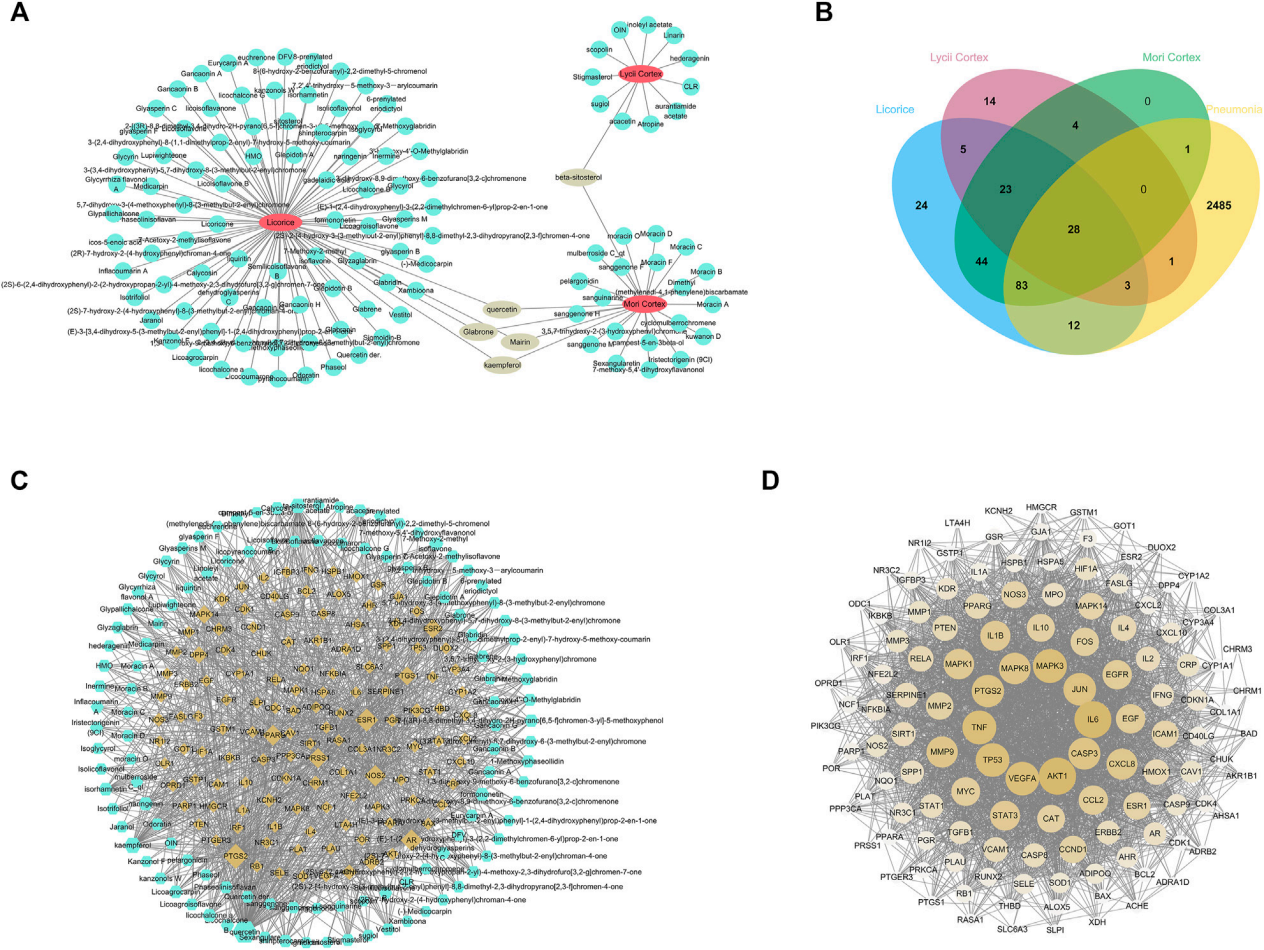
Network pharmacology predicted the active ingredients-target interactions for XBS against pediatric pneumonia. **(A)** The network of Herbs-active ingredients connection. The red nodes represent ingredients in XBS while the blue ones represent active ingredients. **(B)** The venn diagram for ingredients and pneumonia targets. The 128 overlap targets common between the predicted XBS targets and the pneumonia-related targets. **(C)** Network of targets predicted *via* the XBS-active ingredients. Blue nodes represent the active ingredients in XBS, whereas the yellow nodes represent the predicted targets. The edges represent the interaction between compounds and targets. **(D)** PPI network of the XBS ingredients targets against pediatric pneumonia.

To identify the active ingredients of XBS from the TCMSP database, two classical ADME parameters, OB and DL, were used for screening. In this study, a total of 120 active ingredients were recognized in the formula, including 25 in *Mori Cortex*, 12 in *Lycii Cortex* and 88 *in Licorice*. Among the ingredients, five active ingredients (quercetin, mairin, kaempferol, glabrone and beta-sitosterol) were duplicated. The details are summarized in Supplementary Table S2. The herb-active ingredients network was constructed as shown in Figure 1A.

### Construction of Ingredients-Targets-Pneumonia-Related Network of XBS

In this study, the TCMSP database was used to obtain the targets of active ingredients of XBS. A total of 242 potential targets were predicted through these 120 active ingredients above. Next, the pneumonia-related targets were obtained from multisource databases, including OMIM, Genecards, Drugbank, DisgeNET and DigSee, resulting in the retrieval of 2,613 targets. After the construction of the Venn diagram (Figure 1B), 128 common targets were filtered as the key targets between the related targets of XBS and pneumonia, and then a network with 243 nodes and 1,047 edges was constructed (Figure 1C). Among them, quercetin (edges = 107), kaempferol (edges = 41), naringenin (edges = 20), licochalcone A (edges = 18) and isorhamnetin (edges = 18) are the ingredients closely linked to the targets, indicating that flavonoids are probably the most critical ingredients in XBS.

To further explore the underlying pharmacological effects of XBS as a treatment against pediatric pneumonia, the. tsv file obtained from the STRING database with a medium confidence score >0.400 was used to construct the PPI network through Cytoscape software (version 3.6.0) for the 128 common targets. In this PPI network, there were 128 nodes and 2,745 edges in total (Figure 1D). The average node degree was 42.9. The 10 targets with the highest degree were AKT1 (degree = 106), IL6 (degree = 105), TNF (degree = 94), MAPK3 (degree = 94), VEGFA (degree = 93), TP53 (degree = 93), JUN (degree = 89), PTGS2 (degree = 88), CASP3 (degree = 87) and MAPK8 (degree = 83), respectively. These hub genes are most likely involved in various pathogenic processes of pneumonia including inflammatory response, immune suppression, and cell apoptosis.

### Biological Functional Enrichment Analysis of XBS

**FIGURE 2:**
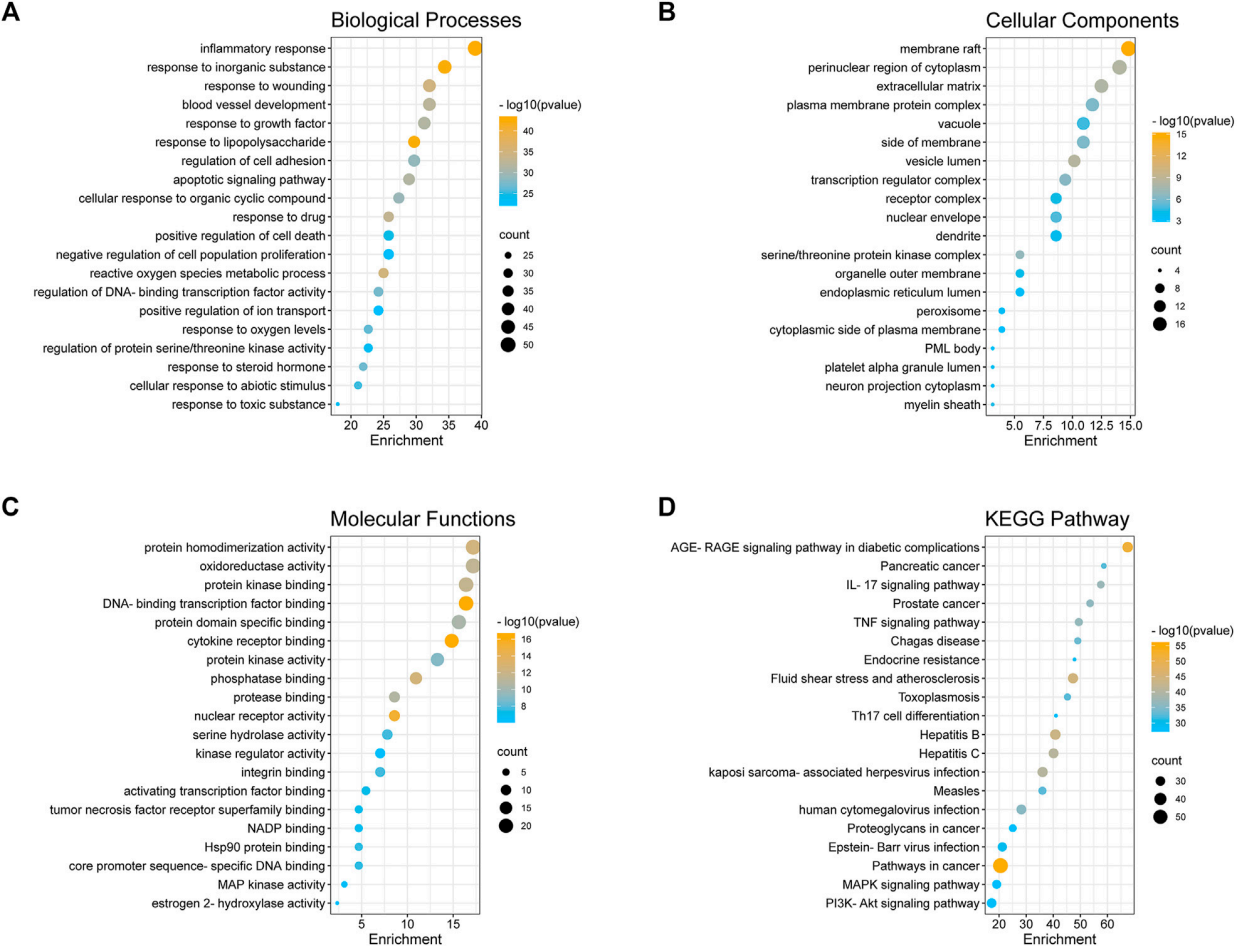
GO enrichment and KEGG pathway analysis of the anti-pneumonia targets of XBS. **(A)** Biological Processes, **(B)** Cellular Components, and **(C)** Molecular Function, **(D)** KEGG Pathway.

In order to elucidate the biological functions of the 128 predicted targets, a GO enrichment analysis was performed. The top 20 enriched biological process terms, molecular function terms, and cellular component terms were determined (Figures 2A–C). The main terms of biological processes were related to the inflammatory response, the response to lipopolysaccharide, the apoptotic signaling pathway, etc. Cellular components enrichment was mainly involved in membrane raft, perinuclear region of cytoplasm, transcription regulator complex, etc. Molecular functions were associated with cytokine receptor binding, DNA-binding transcription factor binding, nuclear receptor activity, etc. All these results indicated that the anti-pediatric pneumonia effect of XBS was strongly linked with the processes involved in its regulatory effect on inflammatory response.

To further confirm the association between the biological functions and target proteins, the top 20 significantly enriched KEGG pathway enrichment of 128 common genes was conducted to show the potential underlying mechanism of XBS for anti-pneumonia based on the log*P* value (Figure 2D, Supplementary Table S3). Although pathways in cancer (hsa05200) involved more targets than others, considering the GO enrichment analysis results, we conjectured that the underlying mechanism of XBS against pediatric pneumonia is principally through the inflammatory-related pathways, such as the IL-17 signaling pathway (hsa04657), the TNF signaling pathway (hsa04668), the PI3K-Akt signaling pathway (hsa04151) and the MAPK signaling pathway (hsa04010), which play important roles in the inflammatory response and cell apoptosis.

### Identification of the Ingredients Characterization of XBS

**FIGURE 3:**
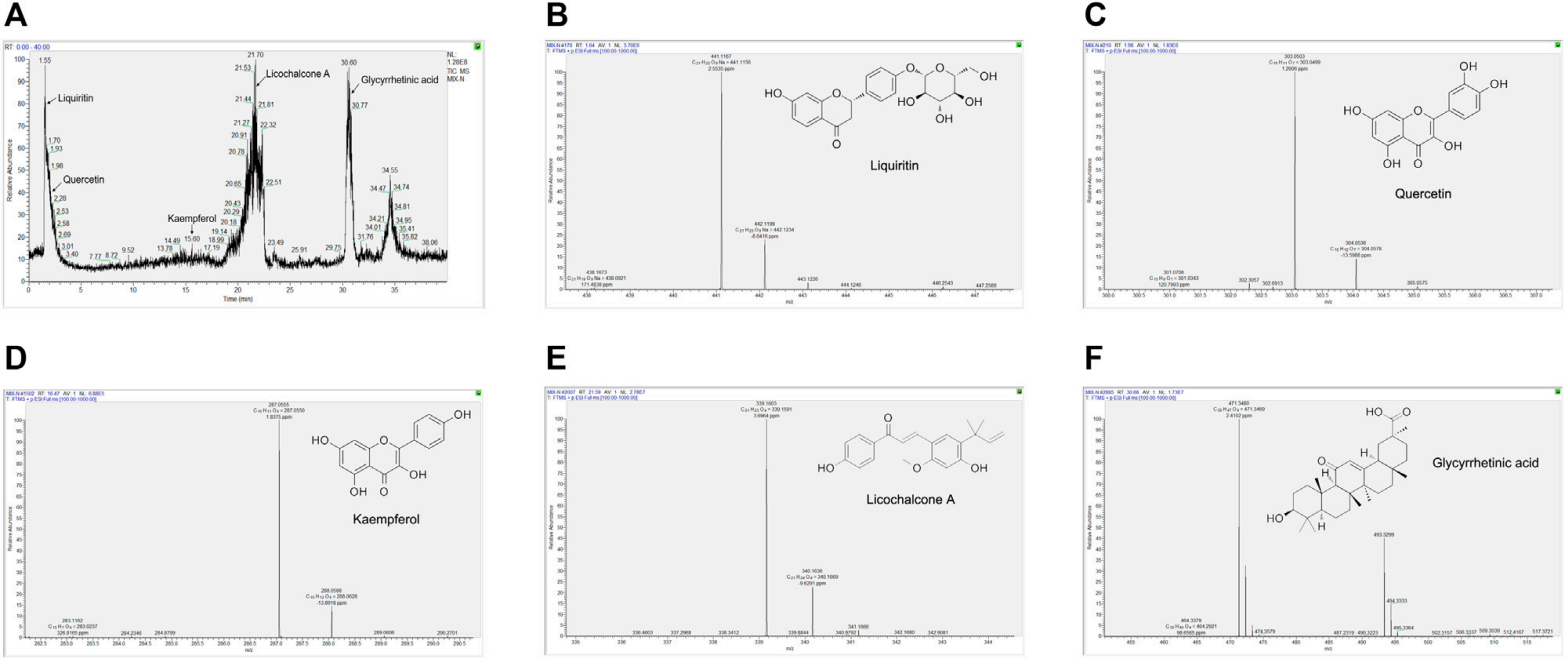
The key ingredients of XBS determined by UPLC-Q/Orbitrap HRMS. Identification No. **(A)** The chromatogram of XBS. **(B)** Liquiritin. **(C)** Quercetin. **(D)** Kaempferol. **(E)** Licochalcone A. **(F)** Glycyrrhetinic acid.

Although the relevant signaling pathways of XBS against pediatric pneumonia based on the active ingredients identified from *Mori Cortex*., *Lycii Cortex*., and *Licorice* were predicted, whether the predicted active ingredients exist in XBS still needs to be verified. Subsequently, the UPLC-Q/Orbitrap HRMS method was adopted for the identification of the bioactive ingredients present in the XBS sample. As shown in Figure 3 and Supplementary Table S4, liquiritin, quercetin, kaempferol and licochalcone A, the most important active ingredients screened in the construction of ingredients-targets-pneumonia-related network of XBS, together with glycyrrhetinic acid, were unequivocally identified *via* X calibur 2.2 software package analysis, verifying the existence of these active ingredients. The results indicated the importance of XBS flavonoids in treating pediatric pneumonia. Reference standards results are shown in Supplementary Figure S1.

### Interactions Between Active Ingredients and Targets

Based on the results above, molecular docking for the 5 HRMS-identified ingredients to selected targets (NF-κB p65, Akt1 and Caspase-3) were performed to investigate the drug-target interaction modes.

**FIGURE 4:**
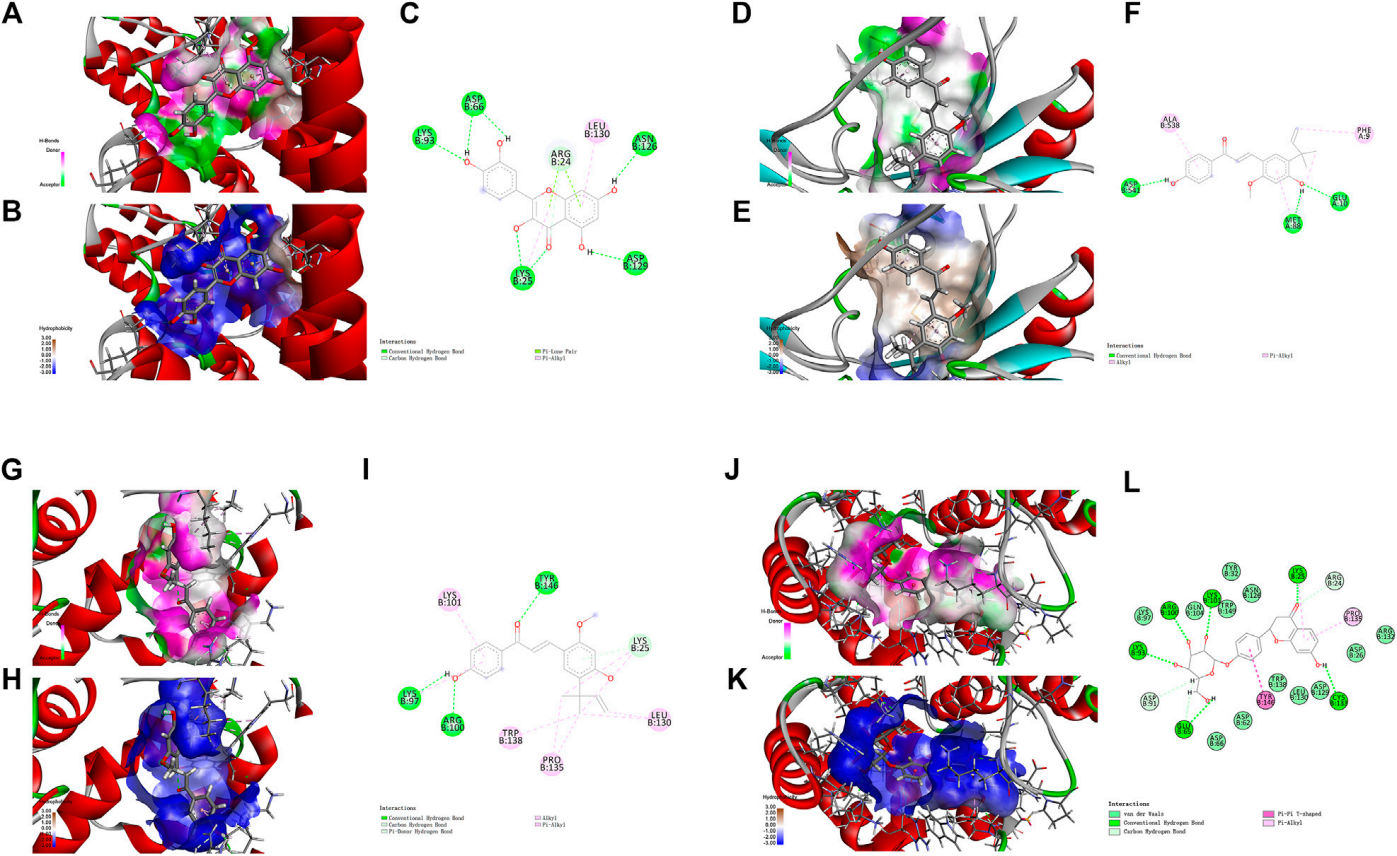
Molecular docking analysis showing bond pattern of NF-κB p65 and quercetin **(A–C)**, kaempferol **(D–F)**, licochalcone A **(G–I)**, liquiritin **(J–L)**. **(A,D,G,J)**: Areas of the donor and acceptor of hydrogen bond (H-bond). **(B,E,H,K)**: Areas of hydrophobicity. **(C,F,I,L)**: Two dimensional patterns of bond.

The nuclear factor κB (NF-κB)/Rel family, such as p65/RelA induces the transcription of a number of inflammatory genes and immune response genes (Siebenlist et al., 1994; Verma et al., 1995). As exhibited in the KEGG pathway enrichment analysis for the targets of XBS, NF-κB p65 is a member of the IL-17 signaling pathway, the TNF signaling pathway, the PI3K-Akt signaling pathway, Th-17 cell differentiation and the MAPK signaling pathway (Supplementary Table S3). For verification, the active ingredients of XBS binding to NF-κB p65 were analyzed with regard to their docking score using a molecular docking method, and those which present a high docking score (total score) might have strong activity. In our study, molecular docking binding to NF-κB p65 provided a total score of 11.9543 for quercetin, 8.6113 for kaempferol, 7.7152 for licochalcone A and 12.7512 for liquiritin (Supplementary Table S5), inferring that these test ingredients may directly interact with NF-κB p65 to regulate the inflammatory response. Figure 4 shows the interaction modes of these ingredients with NF-κB p65: Quercetin interacts with the residues LYS 93, ASP 66, LYS 25, ASN 126 and ASP 129 by means of hydrogen bonding, with the residue ARG 24 through a carbon hydrogen bond, and with the residue LEU 130 *via* hydrophobicity (Figures 4A–C). Kaempferol interacts with the residues ASP 541, MET 88 and GLU 10 by means of a conventional hydrogen bond, and with the residues ALA 538 and PHE 9 *via* hydrophobicity (Figures 4D–F). Licochalcone A interacts with the residues LYS 97, ARG 100 and TYR 146 by means of a conventional hydrogen bond, with the residue LYS 25 through a carbon hydrogen bond, and with the residues LYS 101, TRP 138, PRO 135 and LEU 130 *via* hydrophobicity (Figures 4G–I). Liquiritin interacts with the residues GLU 65, LYS 93, ARG 100, LYS 101, LYS 25 and CYS 133 by means of a conventional hydrogen bond, with the residues ASP 91, GLU 65 and ARG 24 through a carbon-hydrogen bond, with the residues LYS 97, GLN 104, TRP 149, ASN 126, TYR 32, ARG 132, ASP 26, ASP 129, LEU 130, TRP 138, ASP 62 and ASP 66 *via* van der Waals interactions forces, with the residues LYS 25 and PRO 135 *via* hydrophobicity in the form of a π bond, and with the residue TYR 146 through a π-π T-shaped effect. (Figures 4J–L).

Notably, stimulation of the NF-κB pathway promotes apoptosis. One underlying mechanism in this pro-apoptotic activity is nucleolar sequestration of NF-κB p65/RELA (Khandelwal et al., 2011). Thus, we further investigated the ingredients (quercetin and kaempferol) with Akt1 and Caspase-3, the targets closely related to apoptosis. In the present study, molecular binding to Akt1 provided a total score of 7.6115 for quercetin and 10.5769 for kaempferol (Supplementary Table S6). The docking to Caspase-3 gave a total score of 6.2988 for quercetin, and 8.0487 for kaempferol (Supplementary Table S7).

**FIGURE 5:**
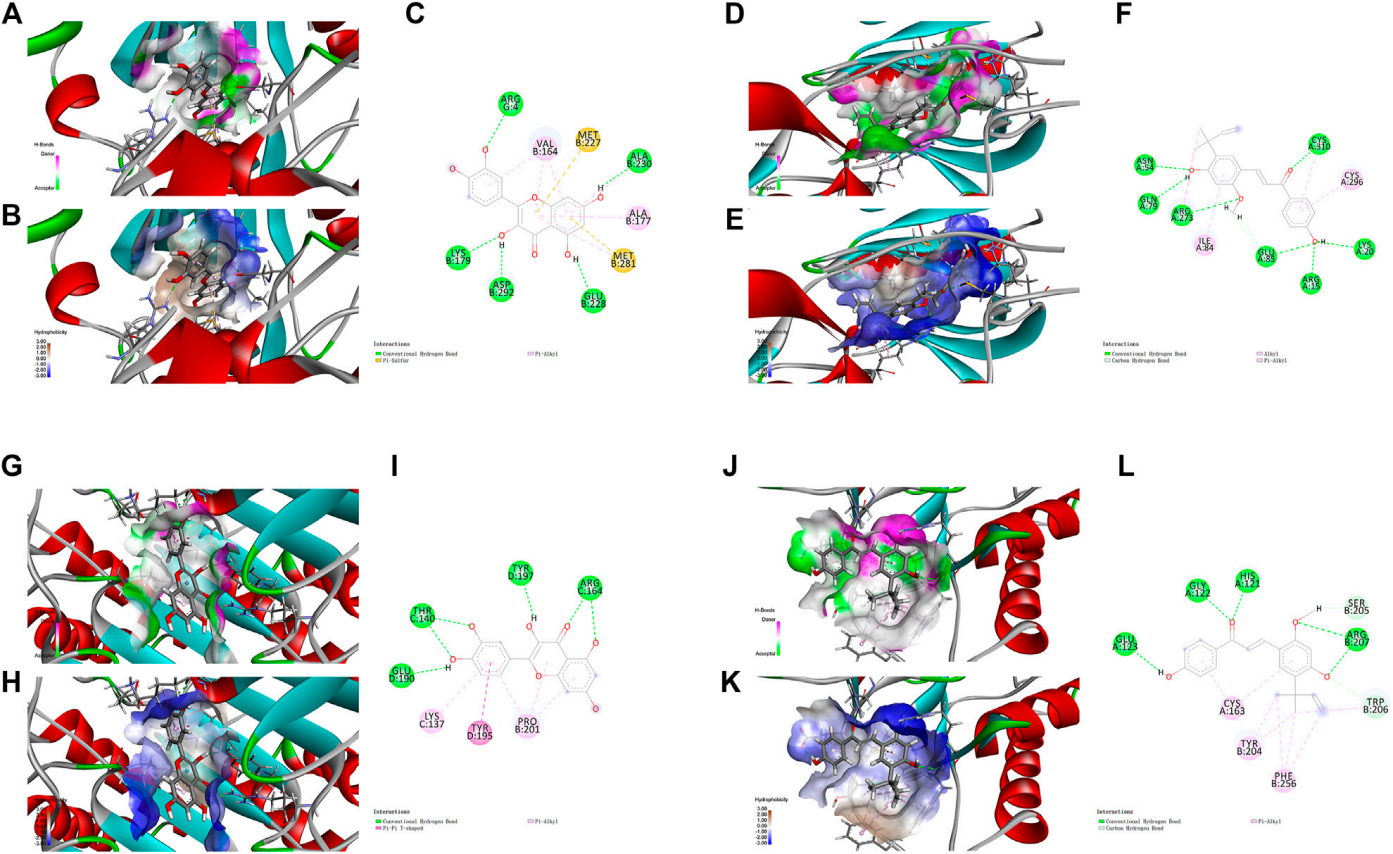
Molecular docking analysis showing bond pattern of Akt1 and quercetin **(A–C)**, kaempferol **(D–F)**; Caspase-3 binding to quercetin **(G–I)**, kaempferol **(J–L)**. **(A,D,G,J)**: Areas of the donor and acceptor of hydrogen bond (H-bond). **(B,E,H,K)**: Areas of hydrophobicity. **(C,F,I,L)**: Two dimensional patterns of bond.

For Akt1, quercetin demonstrated conventional hydrogen bond interactions with the residues ARG 4, LYS 179, ASP 292, GLU 228 and ALA 230, a π-Sulfur type effect with the residues MET 227 and MET 281, and hydrophobicity interactions with the residues VAL 164 and ALA 177, respectively (Figures 5A–C). Kaempferol had conventional hydrogen bond interactions with the residues ASN 54, GLN 79, ARG 273, GLU 85, ARG 15, LYS 20 and CYS 310, a carbon hydrogen bond with the residue GLU 85, and hydrophobicity interactions with the residues ILE 84 and CYS 296, respectively (Figures 5D–F).

For Caspase-3, quercetin had conventional hydrogen bond interactions with the residues GLU 190, THR 140, TYR 197 and ARG 164, a π-π T-shaped effect with the residue TYR 195, and hydrophobicity interactions with the residues LYS 137 and PRO 201, respectively (Figures 5G–I). Kaempferol had conventional hydrogen bond interactions with the residues GLU 123, GLY 122, HIS 121 and ARG 207, a carbon hydrogen bond with the residue SER205, and hydrophobicity interactions with the residues CYS 163, TYR 204 and PHE 256, respectively (Figures 5J–L).

Collectively, the results from molecular docking suggest that the main active ingredients of XBS might directly interact with NF-κB p65, Akt1 and Caspase-3 to inhibit an inflammatory response and cell apoptosis, which were consistent with the results of previous network pharmacological studies. Detailed information about this part of the work is described in Supplementary Tables S5–S7.

In addition, docking bortezomib and triptolide (NF-κB inhibitors) to NF-κB, MK-2206 2HCl (Akt inhibitor) to Akt1 and Z-Devd-fmk (Caspase inhibitor) to Caspase-3 were also performed. As expected, all dockings provided high docking scores. The results are shown in Supplementary Figure S1 and Supplementary Table S8.

### The Dynamics Stability Simulation of Active Ingredients of XBS With NF-κB p65

**FIGURE 6:**
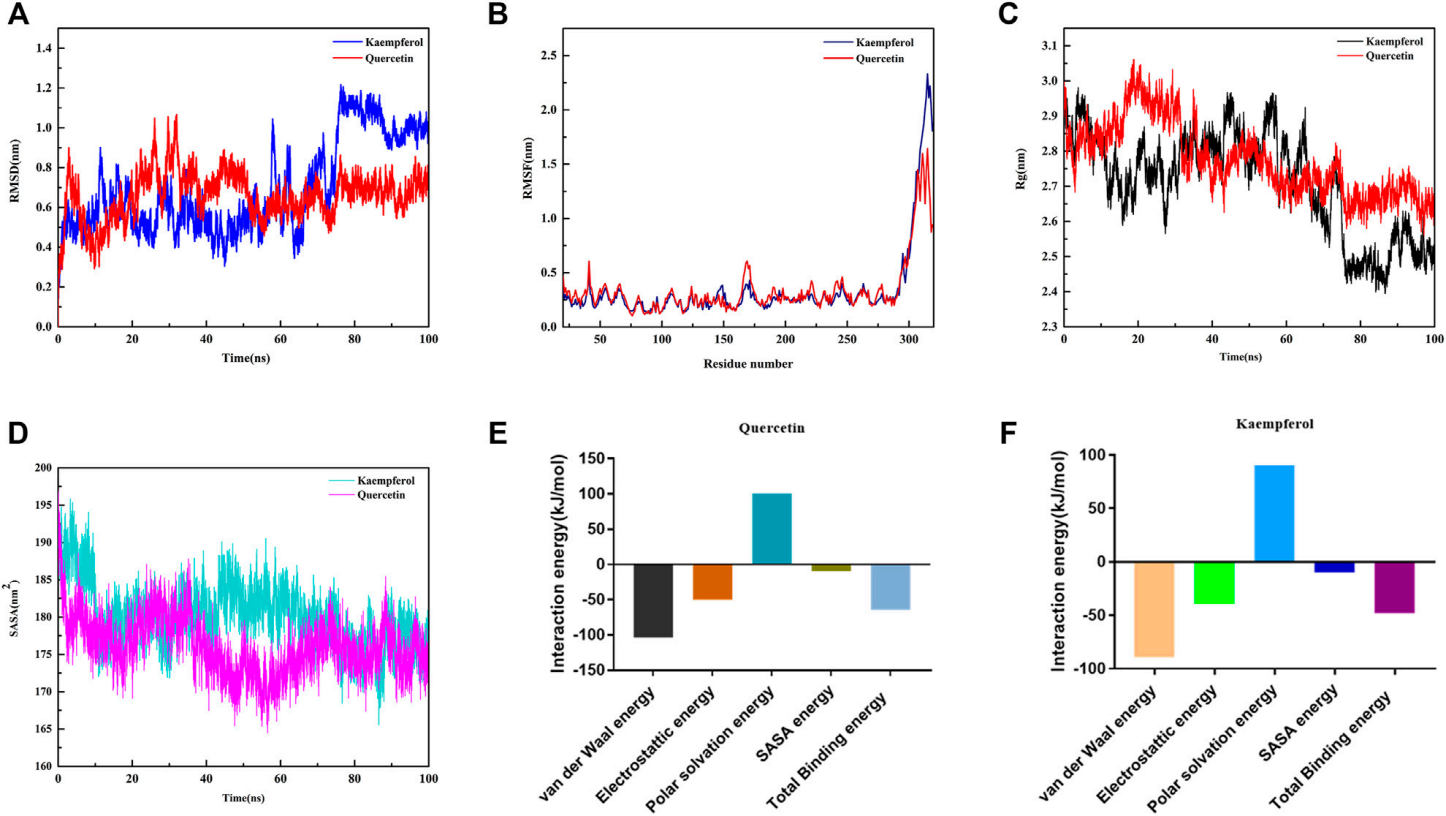
Profiles of molecular dynamics simulations. **(A)** RMSD analysis for the protein-ligand complexes. **(B)** RMSF analysis for the protein-ligand complexes. **(C)** Plot of Rg varies with time. **(D)** Plot of SASA of complexes. **(E)** Binding free energy decomposition of quercetin-NF-κB p65 complex. **(F)** Binding free energy decomposition of kaempferol-NF-κB p65 complex.

On the basis of docking results, quercetin and kaempferol with NF-κB p65 were further selected to perform computer MD simulation study in the hope of understanding their stability in the binding pocket. Of note, the root mean square deviation (RMSD) is an important basis to measure whether the system is stable or not. In this study, the kaempferol-NF-κB p65 system had a sharp rise within 75 ns and tended to balance out the last 5 ns with the average RMSD value was 0.999 ± 0.036. Meanwhile, the quercetin-NF-κB p65 system was in an equilibrium state at 75–100 ns with a smaller average RMSD value (0.695 ± 0.054), indicating that the stability of the system was stronger (Figure 6A). Next, the flexible change in amino acid residues in NF-κB p65 and the root mean square fluctuation (RMSF) value were evaluated. The fluctuations of the two systems in the 295–319 regions were relatively high. Conversely, most of the residues fluctuated at lower values in other regions, suggesting that the binding residues with NF-κB p65 were stable (Figure 6B). Subsequently, as can be seen from the plot of Rg values over time, the overall Rg value of the two systems was in a downward trend, indicating that the stability of the entire system was gradually increasing. Interestingly, the radius of gyration (Rg) value of the quercetin-NF-κB p65 system converged faster and was more stable (Figure 6C). In addition, the solvent-exposed surface area of protein was analyzed by using solvent-accessible surface area (SASA). As the MD simulation progressed, the SASA gradually decreased and stabilized at the 90–100 ns time period with the average values of 176.76 ± 2.15 nm^2^ for kaemferol and 174.69 ± 2.33 nm^2^ for quercetin (Figure 6D). Finally, the results of the binding free energy analysis shown that the van der Waal energy between quercetin and NF-κB p65 was −103.835 kJ/mol, the electrostatic energy was −50.277 kJ/mol, the polar solvation energy was 100.543 kJ/mol, the SASA energy was −10.204 kJ/mol, and the total binding energy was −63.773 kJ (Figure 6E). By contrast, the van der Waal energy of NF-κB p65 with kaempferol was −89.220 kJ/mol, electrostatic energy was −39.310 kJ/mol, polar solvation energy was 90.379 kJ/mol, SASA energy was −10.082 kJ/mol and the total binding energy was −48.233 kJ/mol (Figure 6F). The main contribution of amino acid residues in the binding regions of quercetin and kaempferol to the binding free energy is shown in Table 1 and Table 2. Taken together, all the results confirm that the stability of NF-κB p65, with quercetin and kaempferol, is complex in the process of MD simulation.

**TABLE 1:** Energy contribution of the amino acid residues in quercetin-NF-κB p65 complex (kcal/mol).

Residue	MM energy	Polar energy	APolar energy	Total binding energy
GLU-193	−1.5711	−0.0402	−0.0434	−1.664
LYS-195	−12.1894	14.7148	−0.9486	1.5691
ILE-196	−12.7374	8.3502	−0.5769	−4.9665
CYS-197	−1.9541	0.8429	−0.1067	−1.2177
ASP-217	0.3679	−2.6891	0	−2.321
SER-281	−5.8283	2.1661	−0.025	−3.6935
GLU-282	−14.1846	10.9347	−0.9743	−4.2258
PRO-283	−7.95	7.0112	−0.3111	−1.2523
MET-284	−10.0788	3.5403	−0.9126	−7.4508
GLU-285	−1.5706	0.6285	−0.1225	−1.0671

**TABLE 2:** Energy contribution of the amino acid residues in kaempferol-NF-κB p65 complex (kcal/mol).

Residue	MM energy	Polar energy	APolar energy	Total binding energy
GLU-193	−6.2806	8.7967	−0.204	2.2732
LYS-195	−6.3466	8.0102	−0.4554	1.2122
ILE-196	−7.6339	4.9485	−0.5673	−3.244
VAL-199	−1.9334	0.8377	−0.131	−1.2245
ASP-217	0.1869	−1.7022	−8E-4	−1.5163
MET-284	−5.5091	1.8446	−0.6061	−4.2698

### XBS Blocked LPS-induced Inflammatory Responses in RAW 264.7 Cells

**FIGURE 7:**
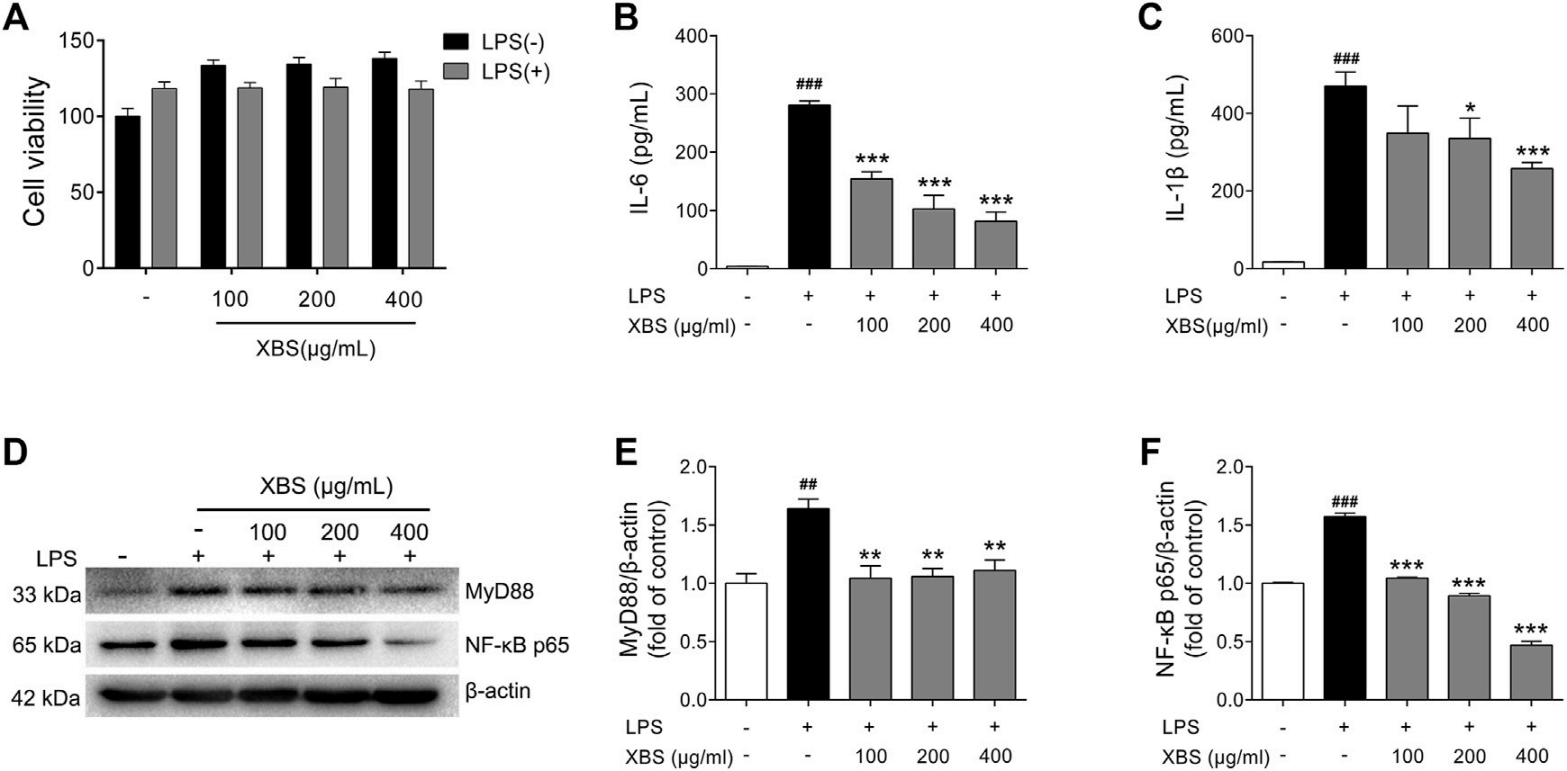
Effects of XBS on inflammatory responses induced by LPS in RAW 264.7 cells. **(A)** Cell viability measured after treatment with XBS alone or XBS in combination with LPS for 24 h. Levels of IL-6 **(B)** and IL-1β **(C)** in culture supernatants were measured using ELISA kits. **(D-F)** Representative western blots showing the protein of MyD88 and NF-κB p65 changes and normalized to β-actin. Similar results were obtained from three independent experiments. The values are presented as means ± SD. ^##^ *p* < 0.01 and ^###^ *p* < 0.001 vs. the control group. ^*^ *p* < 0.05, ^**^ *p* < 0.01 and ^***^ *p* < 0.001 vs. the LPS group.

XBS was predicted to have the ability to regulate NF-κB p65 in above study. The effects of XBS on inflammation and NF-κB p65 activation were then investigated in RAW 264.7 cells using LPS as the inducer. Exposure to LPS results in the activation of myeloid differentiation primary response 88 (MyD88)/NF-κB signaling, leading the dimers (mainly p50/p65) to enter the nucleus and regulate various inflammatory mediators, including IL-6 and IL-1β (Xia et al., 2012). In our study, the results of the CCK-8 assay show that incubation with different dosages of XBS plus LPS was not toxic to RAW264.7 cells (Figure 7A). LPS stimulated the cellular expression of MyD88, NF-κB, and the secretion of both IL-6 and IL-1β as expected, while XBS pre-treatment significantly decreased LPS-induced cytokine production and markedly down-regulated the protein expression of MyD88 and NF-κB p65 (Figures 7B–F), indicating that XBS effectively blocked LPS-induced inflammation in RAW 264.7 cells through inhibiting MyD88/NF-κB signaling.

### XBS Inhibited LPS-induced Cell Apoptosis in RAW 264.7 Cells

**FIGURE 8:**
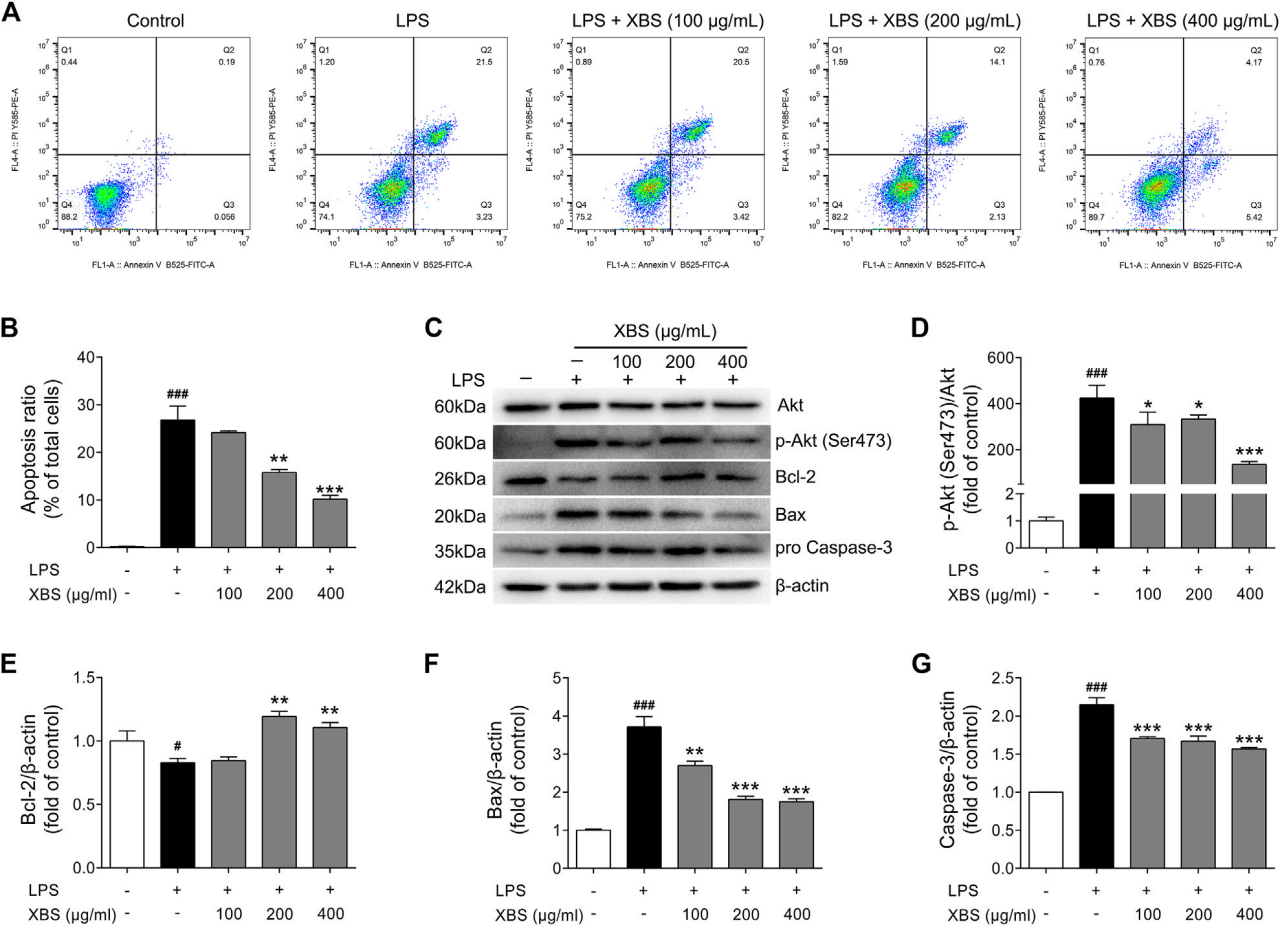
Effects of XBS on cell apoptosis induced by LPS in RAW 264. Seven cells. **(A)** The cells were stained with Annexin V-FITC and PI and the apoptosis rate of cells was analysed by flow cytometry. **(B)** Apoptosis ratio. **(C–G)** Representative Western blots showing the protein of Akt, pAkt (Ser473), Bcl-2, Bax and pro Caspase-3 changes and normalized to β-actin. Similar results were obtained from three independent experiments. The values are presented as means ± SD. ^#^ *p* < 0.05 and ^##^ *p* < 0.001 vs. the control group. ^*^ *p* < 0.05, ^**^ *p* < 0.01 and ^***^ *p* < 0.001 vs. the LPS group.

Studies have shown that NF-κB has a pro-apoptotic role in neutrophils during inflammation (Lawrence et al., 2001; Pan et al., 2008). Nucleolar NF-κB p65/RelA mediates apoptosis by causing cytoplasmic relocalization of nucleophosmin (Khandelwal et al., 2011). XBS, showing the ability to regulate MyD88/NF-κB signaling in RAW 264.7 cells, was then investigated the activity in inhibiting apoptosis during LPS-induced inflammation. AnnexinV-FITC/PI staining and flow cytometry were performed to determine the apoptotic cells rates. As expected, LPS exposure resulted in the number of apoptotic cells. Conversely, with treatment of XBS, LPS-induced cell apoptosis was inhibited in a dose-dependent manner (Figures 8A,B). Furthermore, LPS up-regulated the expression of pro-apoptotic proteins p-Akt (Ser473)/Akt, Bax and Caspase-3, and down-regulated the expression of anti-apoptotic protein Bcl-2, while the treatment with XBS markedly reversed the LPS-induced alteration of the expression of these proteins (Figures 8C–G). The results suggested that the anti-inflammatory effect of XBS in RAW 264.7 cells might be achieved through inhibiting cell apoptosis, and confirmed that the PI3K/Akt signaling pathway predicted by the network pharmacology analysis plays an important role in XBS against pediatric pneumonia.

## Discussion

Pneumonia is a type of acute lower respiratory infection that is common and severe (Quinton et al., 2018). It continues to be the world’s leading infectious cause of death in children worldwide under the age of five. Most deaths occur among children under the age of two (Garenne et al., 1992; Howie and Murdoch, 2019). Although current immunizations are continuously improving to protect against infection sources, mortality from pneumonia remains high. To lessen this persistent mortality, it is critical to explore modalities that can improve outcomes.

Although XBS has been widely used to treat pediatric pneumonia in clinical settings (Zhang et al., 2020), its pharmacological effects have not been reviewed systematically. In the present study, an integrated network pharmacology approach was successfully applied to illuminate the molecular network mechanism of XBS against pediatric pneumonia. Bioinformatics analysis of the 120 bioactive ingredients of XBS and the 128 corresponding pneumonia-related targets revealed that these ingredients are involved in the regulation of inflammation-related pathways (the IL-17 signaling pathway, TNF signaling pathway, PI3K/Akt signaling pathway and MAPK signaling pathway). Additionally, through further component analysis, liquiritin, quercetin, kaempferol, licochalcone A and glycyrrhetinic acid are identified as essential for XBS against pediatric pneumonia.

In the past few years, molecular docking has become an important common component of the drug design and discovery toolbox (Berry et al., 2015). It allows the prediction of molecular interactions that hold together a protein and a ligand in the bound state (Silakari and Kumar, 2021). NF-κB is a pleiotropic transcription factor closely related to biological processes such as inflammatory response, infection, immunity immune regulation, and cell apoptosis (Koay et al., 2002; Khandelwal et al., 2011; Ramos Pittol et al., 2018). A pivotal mediator of innate immune responses is classical signal transduction of NF-κB, whose transcriptional activity requires the Rel-like domain-containing protein (RelA), also known as p65 (Quinton and Mizgerd, 2011). In this study, molecular docking of the identified active ingredients with the anti-pediatric pneumonia targets was carried out to explore the pharmacological targets of XBS. Remarkably, quercetin, kaempferol, licochalcone A and liquiritin, which are shown to be essential for XBS against pediatric pneumonia, showed strong binding affinity to NF-κB p65, a result in accordance with those reported in (Chu et al., 2012; Li et al., 2018; Li and Zhang, 2018; Al Sabaani, 2020), indicating that these active ingredients of XBS directly inhibited NF-κB p65-mediated inflammatory signaling transduction. In fact, NF-κB p65 translocates into the nucleus to activate multiple target genes involved in apoptosis (Khandelwal et al., 2011), while quercetin (Lu et al., 2018) and kaempferol were reported to exert pharmacological effects *via* inhibiting Akt1 activation (Xu et al., 2017). In this study, quercetin and kaempferol exhibited a high affinity to bind with Akt1 and Caspase-3, indicating that the bioactive ingredients of XBS play crucial roles in regulating inflammation and cell apoptosis. Furthermore, MD simulation, one of the effective tools used to check the stability of the protein ligand complex (Majumder and Mandal, 2020), was applied to assess the stability of quercetin and kaempferol to NF-κB p65 in the binding pocket in 100 ns. Interestingly, the MD simulation results are in agreement with the molecular docking results and suggest the favorable stability along with proper interaction during the time, for both bioactive ingredients with the NF-κB p65 protein.

Pneumonia is one of the common respiratory tract inflammatory diseases. Studies have found that increasing neutrophil NF-κB activity contributes to inflammatory damage in the lung (Abraham, 2003). LPS, the cell wall component of Gram-negative bacteria, exposure to the lung elicits the activation of many macrophages and the release of pro-inflammatory cytokines (Jeyaseelan et al., 2004). Macrophages are important cells in inflammatory process and have a critical role in several inflammatory diseases. LPS-stimulated macrophages can activate the translocation of NF-κB p65 protein from the cytoplasm into the nucleus to induce the release of inflammatory cytokines (Hoffmann, et al., 2002; Hayden and Ghosh, 2008; He et al., 2017). Of note, MyD88 is a critical adaptor molecule of Toll-like receptor (TLR) signaling, as well as an important upstream regulatory protein of NF-κB p65 (Lawrence, 2009). MyD88 activation can induce the translocation of downstream mediators NF-κB p65 into the nucleus, leading to pro-inflammatory cytokine production (Deguine and Barton, 2014). In the current study, we explored the anti-inflammation mechanism of XBS treatment on LPS-activated murine alveolar macrophages cell line RAW 264.7. As expected, XBS administration effectively suppressed LPS-induced MyD88 and NF-κB p65 protein expression and reduced the release of IL-1β and IL-6, confirming the ability of XBS to mitigate inflammation.

Furthermore, our study also verified the regulatory effect of XBS on the apoptotic pathway. In the normal resolution of inflammatory reactions, apoptosis is acknowledged to play a crucial role (Oyinloye et al., 2015). The activation of NF-κB signaling is closely related to the expression of apoptosis-associated proteins (Hayden et al., 2006; Lo et al., 2011). LPS stimulation is not only involved in the inflammatory response but also triggers downstream apoptotic pathways, such as Bax, Bcl-2 and Caspase-3 (Song et al., 2014). Bcl-2, an antiapoptotic protein in the outer mitochondrial wall, found to inhibit caspase activity by preventing the release of cytochrome c from the mitochondria and/or binding to the apoptosis-activating factor, has a close connection with intracellular apoptotic signal transduction (Bruey et al., 2007). In contrast, Bax, a proapoptotic protein, promotes cytochrome c release and triggers the activation of Caspase-3, leading to apoptosis (Rossé et al., 1998). Notably, we observed that XBS treatment suppressed apoptosis by up-regulating the expression of Bcl-2 and down-regulating pAkt (Ser437)/Akt, Bax and Caspase-3, which were partly mediated by the inhibition of the PI3K/Akt signaling pathway. Therefore, our experimental results in LPS-induced macrophages confirm the pharmacological effects of XBS in inhibiting inflammation and apoptosis, revealing a mechanism for XBS in treating pediatric pneumonia.

## Conclusion

Collectively, this study uses a scientific approach to predict and verify the targets of XBS anti-pediatric pneumonia. The results suggest that the pharmacological mechanism of XBS might be concerned with the inhibition of the MyD88/NF-κB p65-mediated inflammatory signal transduction and the suppression of PI3K/Akt-mediated apoptosis. Our results provide scientific evidence to support the development and clinical application of XBS in pediatric pneumonia.

## Data Availability

The original contributions presented in the study are included in the article/Supplementary Material, further inquiries can be directed to the corresponding authors.
