# Supplementary figures and images for: An Integrated Pharmacology-Based Strategy to Investigate the Potential Mechanism of Xiebai San in Treating Pediatric Pneumonia
Source: Front Pharmacol. 2022 Feb 14;13:784729. doi: 10.3389/fphar.2022.784729 (PMC8885115; doi:10.3389/fphar.2022.784729)

Original Scans of Immunoblots

FIGURE 7D

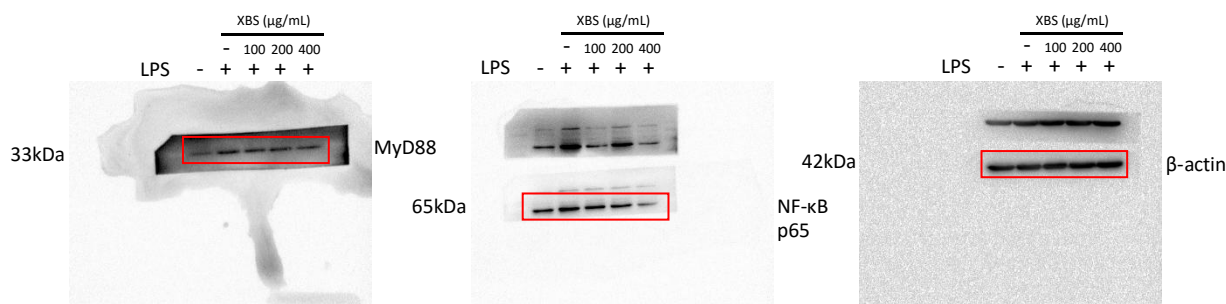

FIGURE 8C

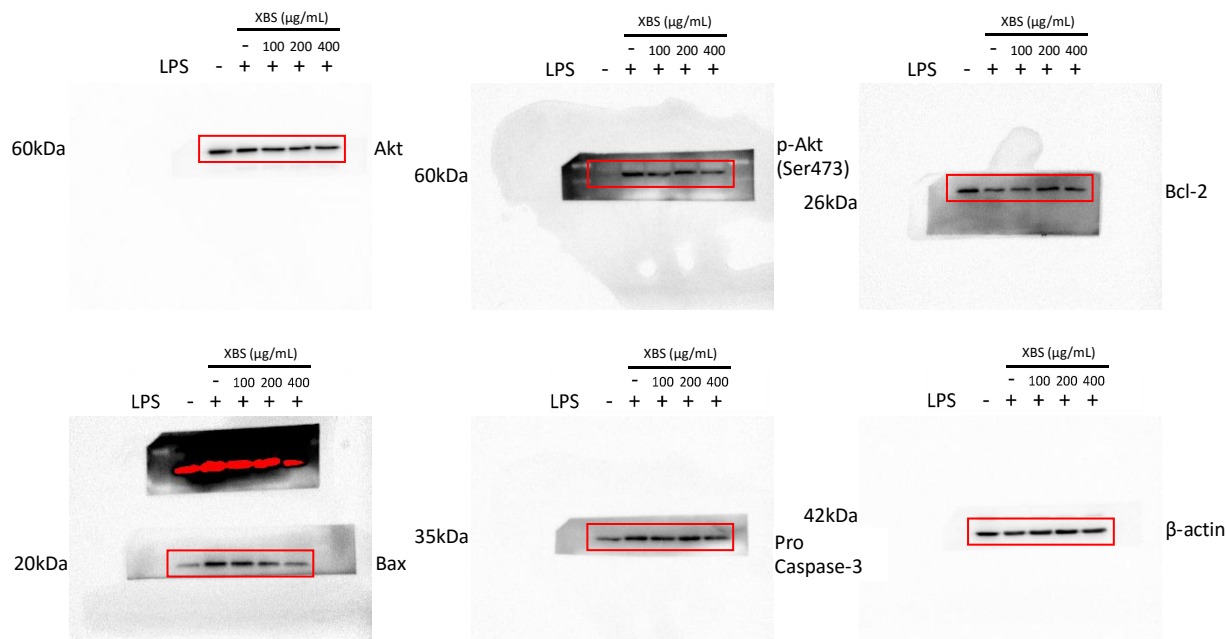

Supplement: Supplementary file 1 [file DataSheet2.pdf]
