# Supplementary material for: An Integrated Pharmacology-Based Strategy to Investigate the Potential Mechanism of Xiebai San in Treating Pediatric Pneumonia
Source: Front Pharmacol. 2022 Feb 14;13:784729. doi: 10.3389/fphar.2022.784729 (PMC8885115; doi:10.3389/fphar.2022.784729)
Supplement: Supplementary file 2 [file DataSheet1.docx]

**Supplemental Material**

**

**

**SUPPLEMENTARY FIGURE 1** | Reference standards determined by UPLC-Q/Orbitrap HRMS. Identification No.: (A) Quercetin. (B) Isorhamnetin. (C) Kaempferol. (D) Glycyrrhetinic acid. (E) Naringenin. (F) Licochalcone A. (G) Liquiritin.

**
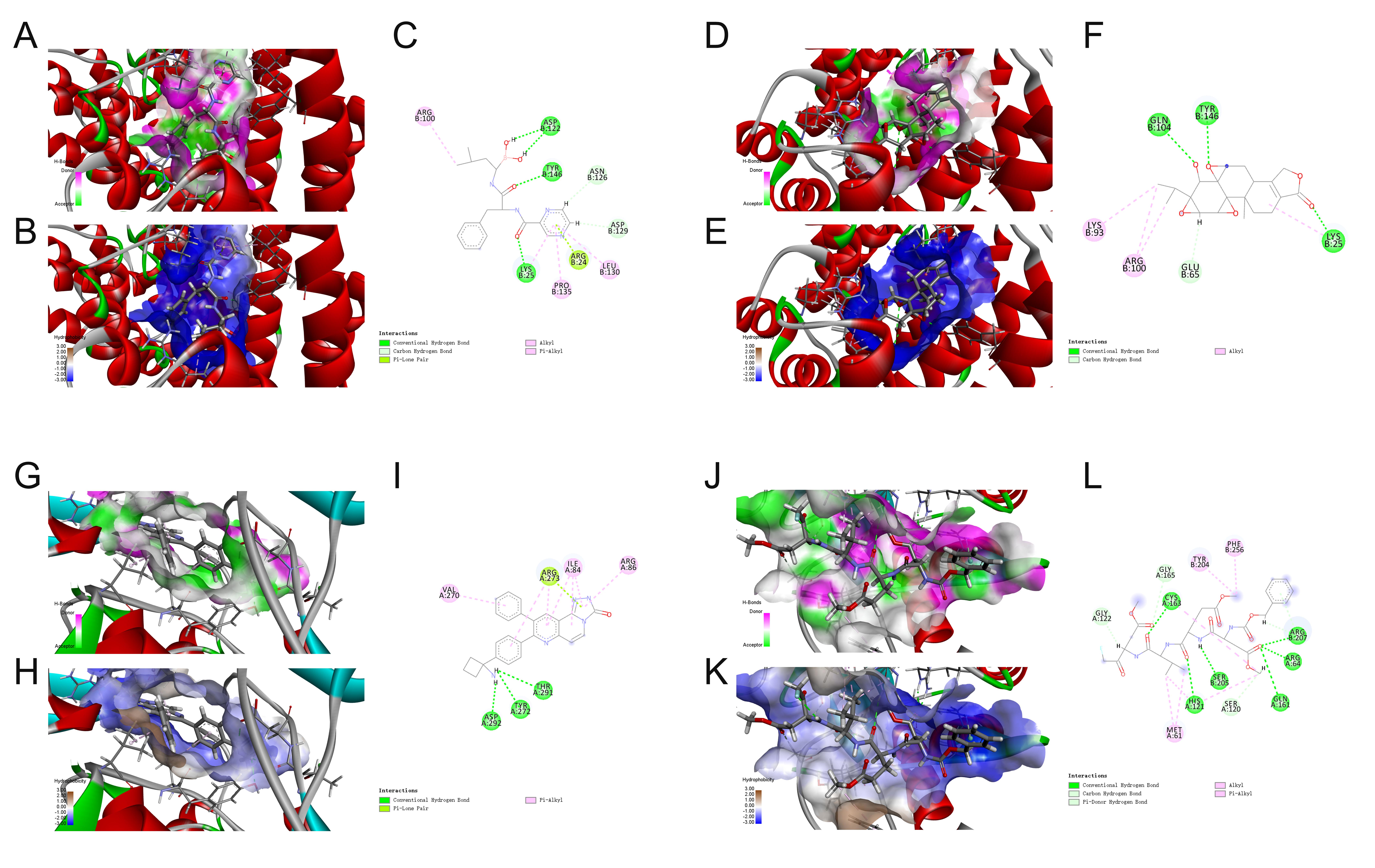
**

**SUPPLEMENTARY FIGURE 2 |** Molecular docking analysis showing the interaction modes of NF κB p65 with bortezomib (A, B and C) or triptolide (D, E and F), Akt1 with MK-2206 2HCl (G, H and I), and Caspase-3 with Z-Devd-fmk (J, K and L). (A), (D), (G) and (J): Areas of the donor and acceptor of hydrogen bond (H-bond). (B), (E), (H) and (K): Areas of hydrophobicity. (C), (F), (I) and (L): Two dimensional patterns of bond.

**SUPPLEMENTARY TABLE 1 |** PDB_ID of target.

| **Target** | **Number** | **PDB_ID** |
| --- | --- | --- |
| NF κB p65 | 1 | 3NR1 |
|  | 2 | 5URN |
|  | 3 | 6QHM |
|  |  |  |
| Akt1 | 1 | 3D0E |
|  | 2 | 6BUU |
|  | 3 | 6HHI |
|  |  |  |
| Caspase-3 | 1 | 2CNO |
|  | 2 | 3GJR |
|  | 3 | 3KJF |

**SUPPLEMENTARY TABLE 2 |** Active ingredients of XBS.

| **MOL ID** | **Molecule Name** | **OB (%)** | **DL** | **Source** |
| --- | --- | --- | --- | --- |
| MOL000098 | quercetin | 46.43 | 0.28 | *Mori Cortex, Licorice* |
| MOL000211 | Mairin | 55.38 | 0.78 | *Mori Cortex, Licorice* |
| MOL000239 | Jaranol | 50.83 | 0.29 | *Licorice* |
| MOL000296 | hederagenin | 36.91 | 0.75 | *Lycii Cortex* |
| MOL000354 | isorhamnetin | 49.6 | 0.31 | *Licorice* |
| MOL000358 | beta-sitosterol | 36.91 | 0.75 | *Mori Cortex, Lycii Cortex* |
| MOL000359 | sitosterol | 36.91 | 0.75 | *Licorice* |
| MOL000392 | formononetin | 69.67 | 0.21 | *Licorice* |
| MOL000417 | Calycosin | 47.75 | 0.24 | *Licorice* |
| MOL000422 | kaempferol | 41.88 | 0.24 | *Mori Cortex, Licorice* |
| MOL000449 | Stigmasterol | 43.83 | 0.76 | *Lycii Cortex* |
| MOL000497 | licochalcone a | 40.79 | 0.29 | *Licorice* |
| MOL000500 | Vestitol | 74.66 | 0.21 | *Licorice* |
| MOL000554 | gallic acid-3-O-(6'-O-galloyl)-glucoside | 30.25 | 0.67 | *Mori Cortex* |
| MOL000953 | CLR | 37.87 | 0.68 | *Lycii Cortex* |
| MOL001004 | pelargonidin | 37.99 | 0.21 | *Mori Cortex* |
| MOL001474 | sanguinarine | 37.81 | 0.86 | *Mori Cortex* |
| MOL001484 | Inermine | 75.18 | 0.54 | *Licorice* |
| MOL001552 | OIN | 45.97 | 0.19 | *Lycii Cortex* |
| MOL001645 | Linoleyl acetate | 42.1 | 0.2 | *Lycii Cortex* |
| MOL001689 | acacetin | 34.97 | 0.24 | *Lycii Cortex* |
| MOL001790 | Linarin | 39.84 | 0.71 | *Lycii Cortex* |
| MOL001792 | DFV | 32.76 | 0.18 | *Licorice* |
| MOL002218 | scopolin | 56.45 | 0.39 | *Lycii Cortex* |
| MOL002219 | Atropine | 34.53 | 0.21 | *Lycii Cortex* |
| MOL002222 | sugiol | 36.11 | 0.28 | *Lycii Cortex* |
| MOL002224 | aurantiamide acetate | 58.38 | 0.59 | *Lycii Cortex* |
| MOL002228 | Kulactone | 45.44 | 0.82 | *Lycii Cortex* |
| MOL002311 | Glycyrol | 90.78 | 0.67 | *Licorice* |
| MOL002514 | Sexangularetin | 62.86 | 0.3 | *Mori Cortex* |
| MOL002565 | Medicarpin | 49.22 | 0.34 | *Licorice* |
| MOL003656 | Lupiwighteone | 51.64 | 0.37 | *Licorice* |
| MOL003758 | Iristectorigenin (9CI) | 71.55 | 0.34 | *Mori Cortex* |
| MOL003856 | Moracin B | 55.85 | 0.23 | *Mori Cortex* |
| MOL003857 | Moracin C | 82.13 | 0.29 | *Mori Cortex* |
| MOL003858 | Moracin D | 60.93 | 0.38 | *Mori Cortex* |
| MOL003860 | Moracin F | 53.81 | 0.23 | *Mori Cortex* |
| MOL003896 | 7-Methoxy-2-methyl isoflavone | 42.56 | 0.2 | *Licorice* |
| MOL004328 | naringenin | 59.29 | 0.21 | *Licorice* |
| MOL004805 | (2S)-2-[4-hydroxy-3-(3-methylbut-2-enyl)phenyl]-8,8-dimethyl-2,3-dihydropyrano[2,3-f]chromen-4-one | 31.79 | 0.72 | *Licorice* |
| MOL004806 | euchrenone | 30.29 | 0.57 | *Licorice* |
| MOL004808 | glyasperin B | 65.22 | 0.44 | *Licorice* |
| MOL004810 | glyasperin F | 75.84 | 0.54 | *Licorice* |
| MOL004811 | Glyasperin C | 45.56 | 0.4 | *Licorice* |
| MOL004814 | Isotrifoliol | 31.94 | 0.42 | *Licorice* |
| MOL004815 | (E)-1-(2,4-dihydroxyphenyl)-3-(2,2-dimethylchromen-6-yl)prop-2-en-1-one | 39.62 | 0.35 | *Licorice* |
| MOL004820 | kanzonols W | 50.48 | 0.52 | *Licorice* |
| MOL004824 | (2S)-6-(2,4-dihydroxyphenyl)-2-(2-hydroxypropan-2-yl)-4-methoxy-2,3-dihydrofuro[3,2-g]chromen-7-one | 60.25 | 0.63 | *Licorice* |
| MOL004827 | Semilicoisoflavone B | 48.78 | 0.55 | *Licorice* |
| MOL004828 | Glepidotin A | 44.72 | 0.35 | *Licorice* |
| MOL004829 | Glepidotin B | 64.46 | 0.34 | *Licorice* |
| MOL004833 | Phaseolinisoflavan | 32.01 | 0.45 | *Licorice* |
| MOL004835 | Glypallichalcone | 61.6 | 0.19 | *Licorice* |
| MOL004838 | 8-(6-hydroxy-2-benzofuranyl)-2,2-dimethyl-5-chromenol | 58.44 | 0.38 | *Licorice* |
| MOL004841 | Licochalcone B | 76.76 | 0.19 | *Licorice* |
| MOL004848 | licochalcone G | 49.25 | 0.32 | *Licorice* |
| MOL004849 | 3-(2,4-dihydroxyphenyl)-8-(1,1-dimethylprop-2-enyl)-7-hydroxy-5-methoxy-coumarin | 59.62 | 0.43 | *Licorice* |
| MOL004855 | Licoricone | 63.58 | 0.47 | *Licorice* |
| MOL004856 | Gancaonin A | 51.08 | 0.4 | *Licorice* |
| MOL004857 | Gancaonin B | 48.79 | 0.45 | *Licorice* |
| MOL004860 | licorice glycoside E | 32.89 | 0.27 | *Licorice* |
| MOL004863 | 3-(3,4-dihydroxyphenyl)-5,7-dihydroxy-8-(3-methylbut-2-enyl)chromone | 66.37 | 0.41 | *Licorice* |
| MOL004864 | 5,7-dihydroxy-3-(4-methoxyphenyl)-8-(3-methylbut-2-enyl)chromone | 30.49 | 0.41 | *Licorice* |
| MOL004866 | 2-(3,4-dihydroxyphenyl)-5,7-dihydroxy-6-(3-methylbut-2-enyl)chromone | 44.15 | 0.41 | *Licorice* |
| MOL004879 | Glycyrin | 52.61 | 0.47 | *Licorice* |
| MOL004882 | Licocoumarone | 33.21 | 0.36 | *Licorice* |
| MOL004883 | Licoisoflavone | 41.61 | 0.42 | *Licorice* |
| MOL004884 | Licoisoflavone B | 38.93 | 0.55 | *Licorice* |
| MOL004885 | licoisoflavanone | 52.47 | 0.54 | *Licorice* |
| MOL004891 | shinpterocarpin | 80.3 | 0.73 | *Licorice* |
| MOL004898 | (E)-3-[3,4-dihydroxy-5-(3-methylbut-2-enyl)phenyl]-1-(2,4-dihydroxyphenyl)prop-2-en-1-one | 46.27 | 0.31 | *Licorice* |
| MOL004903 | liquiritin | 65.69 | 0.74 | *Licorice* |
| MOL004904 | licopyranocoumarin | 80.36 | 0.65 | *Licorice* |
| MOL004905 | 3,22-Dihydroxy-11-oxo-delta(12)-oleanene-27-alpha-methoxycarbonyl-29-oic acid | 34.32 | 0.55 | *Licorice* |
| MOL004907 | Glyzaglabrin | 61.07 | 0.35 | *Licorice* |
| MOL004908 | Glabridin | 53.25 | 0.47 | *Licorice* |
| MOL004910 | Glabranin | 52.9 | 0.31 | *Licorice* |
| MOL004911 | Glabrene | 46.27 | 0.44 | *Licorice* |
| MOL004912 | Glabrone | 52.51 | 0.5 | *Mori Cortex, Licorice* |
| MOL004913 | 1,3-dihydroxy-9-methoxy-6-benzofurano[3,2-c]chromenone | 48.14 | 0.43 | *Licorice* |
| MOL004914 | 1,3-dihydroxy-8,9-dimethoxy-6-benzofurano[3,2-c]chromenone | 62.9 | 0.53 | *Licorice* |
| MOL004915 | Eurycarpin A | 43.28 | 0.37 | *Licorice* |
| MOL004917 | glycyroside | 37.25 | 0.79 | *Licorice* |
| MOL004924 | (-)-Medicocarpin | 40.99 | 0.95 | *Licorice* |
| MOL004935 | Sigmoidin-B | 34.88 | 0.41 | *Licorice* |
| MOL004941 | (2R)-7-hydroxy-2-(4-hydroxyphenyl)chroman-4-one | 71.12 | 0.18 | *Licorice* |
| MOL004945 | (2S)-7-hydroxy-2-(4-hydroxyphenyl)-8-(3-methylbut-2-enyl)chroman-4-one | 36.57 | 0.32 | *Licorice* |
| MOL004948 | Isoglycyrol | 44.7 | 0.84 | *Licorice* |
| MOL004949 | Isolicoflavonol | 45.17 | 0.42 | *Licorice* |
| MOL004957 | HMO | 38.37 | 0.21 | *Licorice* |
| MOL004959 | 1-Methoxyphaseollidin | 69.98 | 0.64 | *Licorice* |
| MOL004961 | Quercetin der. | 46.45 | 0.33 | *Licorice* |
| MOL004966 | 3'-Hydroxy-4'-O-Methylglabridin | 43.71 | 0.57 | *Licorice* |
| MOL004974 | 3'-Methoxyglabridin | 46.16 | 0.57 | *Licorice* |
| MOL004978 | 2-[(3R)-8,8-dimethyl-3,4-dihydro-2H-pyrano[6,5-f]chromen-3-yl]-5-methoxyphenol | 36.21 | 0.52 | *Licorice* |
| MOL004980 | Inflacoumarin A | 39.71 | 0.33 | *Licorice* |
| MOL004985 | icos-5-enoic acid | 30.7 | 0.2 | *Licorice* |
| MOL004988 | Kanzonol F | 32.47 | 0.89 | *Licorice* |
| MOL004989 | 6-prenylated eriodictyol | 39.22 | 0.41 | *Licorice* |
| MOL004990 | 7,2',4'-trihydroxy－5-methoxy-3－arylcoumarin | 83.71 | 0.27 | *Licorice* |
| MOL004991 | 7-Acetoxy-2-methylisoflavone | 38.92 | 0.26 | *Licorice* |
| MOL004993 | 8-prenylated eriodictyol | 53.79 | 0.4 | *Licorice* |
| MOL004996 | gadelaidic acid | 30.7 | 0.2 | *Licorice* |
| MOL005000 | Gancaonin G | 60.44 | 0.39 | *Licorice* |
| MOL005001 | Gancaonin H | 50.1 | 0.78 | *Licorice* |
| MOL005003 | Licoagrocarpin | 58.81 | 0.58 | *Licorice* |
| MOL005007 | Glyasperins M | 72.67 | 0.59 | *Licorice* |
| MOL005008 | Glycyrrhiza flavonol A | 41.28 | 0.6 | *Licorice* |
| MOL005012 | Licoagroisoflavone | 57.28 | 0.49 | *Licorice* |
| MOL005013 | 18α-hydroxyglycyrrhetic acid | 41.16 | 0.71 | *Licorice* |
| MOL005016 | Odoratin | 49.95 | 0.3 | *Licorice* |
| MOL005017 | Phaseol | 78.77 | 0.58 | *Licorice* |
| MOL005018 | Xambioona | 54.85 | 0.87 | *Licorice* |
| MOL005020 | dehydroglyasperins C | 53.82 | 0.37 | *Licorice* |
| MOL005043 | campest-5-en-3beta-ol | 37.58 | 0.71 | *Mori Cortex* |
| MOL009653 | Cycloeucalenol | 39.73 | 0.79 | *Mori Cortex* |
| MOL012681 | Dimethyl (methylenedi-4,1-phenylene)biscarbamate | 50.84 | 0.26 | *Mori Cortex* |
| MOL012686 | 7-methoxy-5,4'-dihydroxyflavanonol | 51.72 | 0.26 | *Mori Cortex* |
| MOL012689 | cyclomulberrochromene | 36.79 | 0.87 | *Mori Cortex* |
| MOL012692 | kuwanon D | 31.09 | 0.8 | *Mori Cortex* |
| MOL012714 | Moracin A | 64.39 | 0.23 | *Mori Cortex* |
| MOL012717 | moracin M-6,3'-di-O-β-D-glucopyranoside | 37.81 | 0.74 | *Mori Cortex* |
| MOL012719 | moracin O | 62.33 | 0.44 | *Mori Cortex* |
| MOL012726 | mulberrofuran G | 92.19 | 0.24 | *Mori Cortex* |
| MOL012735 | mulberroside C_qt | 71.39 | 0.46 | *Mori Cortex* |
| MOL012743 | resveratrol-3,4'-di-O-β-D-glucopyranoside | 35.08 | 0.76 | *Mori Cortex* |
| MOL012749 | sanggenone B | 115.44 | 0.3 | *Mori Cortex* |
| MOL012753 | sanggenone F | 62.42 | 0.54 | *Mori Cortex* |
| MOL012755 | sanggenone H | 37.5 | 0.53 | *Mori Cortex* |
| MOL012760 | sanggenone M | 68.29 | 0.85 | *Mori Cortex* |
| MOL012800 | 3,5,7-trihydroxy-2-(3-hydroxyphenyl)chromone | 59.71 | 0.24 | *Mori Cortex* |

**SUPPLEMENTARY TABLE 3 |** Annotation of KEGG pathways.

| **Term ID** | **Description** | **Count** | **P value** | **Gene Name** |
| --- | --- | --- | --- | --- |
| hsa05200 | Pathways in cancer | 54 | 7.86E-57 | *AKT1, AKR1B1, FASLG, AR, BAD, BAX, CCND1, BCL2, CASP3, CASP8, CASP9, CDK4, CDKN1A, CHUK, NQO1, EGF, EGFR, ERBB2, ESR1, ESR2, FOS, GSTM1, GSTP1, HIF1A, HMOX1, IFNG, IKBKB, IL2, IL4, IL6, CXCL8, JUN, MMP1, MMP2, MMP9, MYC, NFE2L2, NFKBIA, NOS2, PPARG, PRKCA, MAPK1, MAPK3, MAPK8, PTEN, PTGER3, PTGS2, RB1, RELA, STAT1, STAT3, TGFB1, TP53, VEGFA* |
| hsa04933 | AGE-RAGE signaling pathway in diabetic complications | 33 | 2.25E-52 | *AKT1, BAX, CCND1, BCL2, CASP3, CDK4, COL1A1, COL3A1, MAPK14, F3, ICAM1, IL1A, IL1B, IL6, CXCL8, JUN, MMP2, NOS3, SERPINE1, PRKCA, MAPK1, MAPK3, MAPK8, RELA, CCL2, SELE, STAT1, STAT3, TGFB1, THBD, TNF, VCAM1, VEGFA* |
| hsa05418 | Fluid shear stress and atherosclerosis | 32 | 3.80E-45 | *AKT1, BCL2, CAV1, CHUK, MAPK14, NQO1, FOS, GSTM1, GSTP1, HMOX1, ICAM1, IFNG, IKBKB, IL1A, IL1B, JUN, KDR, MMP2, MMP9, NFE2L2, NOS3, PLAT, MAPK8, RELA, CCL2, SELE, THBD, TNF, TP53, VCAM1, VEGFA, NCF1* |
| hsa05161 | Hepatitis B | 33 | 3.03E-44 | *AKT1, FASLG, BAD, BAX, CCND1, BCL2, CASP3, CASP8, CASP9, CDK4, CDKN1A, CHUK, MAPK14, FOS, IKBKB, IL6, CXCL8, JUN, MMP9, MYC, NFKBIA, PRKCA, MAPK1, MAPK3, MAPK8, PTEN, RB1, RELA, STAT1, STAT3, TGFB1, TNF, TP53* |
| hsa05160 | Hepatitis C | 31 | 2.66E-41 | *AKT1, FASLG, BAD, BAX, CCND1, CASP3, CASP8, CASP9, CDK4, CDKN1A, CHUK, MAPK14, EGF, EGFR, IFNG, IKBKB, CXCL8, CXCL10, IRF1, MYC, NFKBIA, PPARA, MAPK1, MAPK3, MAPK8, RB1, RELA, STAT1, STAT3, TNF, TP53* |
| hsa05167 | kaposi sarcoma-associated herpesvirus infection | 32 | 4.68E-41 | *AKT1, BAX, CCND1, CASP3, CASP8, CASP9, CDK4, CDKN1A, CHUK, MAPK14, FOS, CXCL2, HIF1A, ICAM1, IKBKB, IL6, CXCL8, JUN, MYC, NFKBIA, PIK3CG, PPP3CA, MAPK1, MAPK3, MAPK8, PTGS2, RB1, RELA, STAT1, STAT3, TP53, VEGFA* |
| hsa04657 | IL-17 signaling pathway | 25 | 1.14E-37 | *CASP3, CASP8, CHUK, MAPK14, FOS, CXCL2, IFNG, IKBKB, IL1B, IL4, IL6, CXCL8, CXCL10, JUN, MMP1, MMP3, MMP9, NFKBIA, MAPK1, MAPK3, MAPK8, PTGS2, RELA, CCL2, TNF* |
| hsa04668 | TNF signaling pathway | 26 | 3.10E-37 | *AKT1, CASP3, CASP8, CHUK, MAPK14, FOS, CXCL2, ICAM1, IKBKB, IL1B, IL6, CXCL10, IRF1, JUN, MMP3, MMP9, NFKBIA, MAPK1 ,MAPK3, MAPK8, PTGS2, RELA, CCL2, SELE, TNF, VCAM1* |
| hsa05215 | Prostate cancer | 25 | 8.51E-37 | *AKT1, AKR1B1, AR, BAD, CCND1, BCL2, CASP9, CDKN1A, CHUK, EGF, EGFR, ERBB2, GSTP1, IKBKB, MMP3, MMP9, NFKBIA, PLAT, PLAU, MAPK1, MAPK3, PTEN, RB1, RELA, TP53* |
| hsa05163 | human cytomegalovirus infection | 31 | 2.73E-36 | *AKT1, FASLG, BAX, CCND1, CASP3, CASP8, CASP9, CDK4, CDKN1A, CHUK, MAPK14, EGFR, IKBKB, IL1B, IL6, CXCL8, MYC, NFKBIA, PPP3CA, PRKCA, MAPK1, MAPK3, PTGER3, PTGS2, RB1, RELA, CCL2, STAT3, TNF, TP53, VEGFA* |
| hsa05142 | Chagas disease | 24 | 2.62E-34 | *AKT1, FASLG, CASP8, CHUK, MAPK14, FOS, IFNG, IKBKB, IL1B, IL2, IL6, CXCL8, IL10, JUN, NFKBIA, NOS2, SERPINE1, MAPK1, MAPK3, MAPK8, RELA, CCL2, TGFB1, TNF* |
| hsa05212 | Pancreatic cancer | 22 | 2.02E-33 | *AKT1, BAD, BAX, CCND1, CASP9, CDK4, CDKN1A, CHUK, EGF, EGFR, ERBB2, IKBKB, MAPK1, MAPK3, MAPK8, RB1, RELA, STAT1, STAT3, TGFB1, TP53, VEGFA* |
| hsa05145 | Toxoplasmosis | 24 | 2.23E-33 | *AKT1, ALOX5, BAD, BCL2, CASP3, CASP8, CASP9, CD40LG, CHUK, MAPK14, IFNG, IKBKB, IL10, NFKBIA, NOS2, PIK3CG, MAPK1, MAPK3, MAPK8, RELA, STAT1, STAT3, TGFB1, TNF* |
| hsa05162 | Measles | 26 | 2.49E-33 | *AKT1, FASLG, BAD, BAX, CCND1, BCL2, CASP3, CASP8, CASP9, CDK4, CHUK, FOS, IFNG, IKBKB, IL1A, IL1B, IL2, IL4, IL6, JUN, NFKBIA, MAPK8, RELA, STAT1, STAT3, TP53* |
| hsa05169 | Epstein-Barr virus infection | 29 | 3.11E-30 | *AKT1, BAX, CCND1, BCL2, CASP3, CASP8, CASP9, CDK1, CDK4, CDKN1A, CHUK, MAPK14, HSPB1, ICAM1, IFNG IKBKB, IL6, IL10, CXCL10, JUN, MYC, NFKBIA, MAPK8, RB1, RELA, STAT1, STAT3, TNF, TP53* |
| hsa01522 | Endocrine resistance | 21 | 7.70E-30 | *AKT1, BAD, BAX, CCND1, BCL2, CDK4, CDKN1A, MAPK14, EGFR, ERBB2, ESR1, ESR2, FOS, JUN, MMP2, MMP9, MAPK1, MAPK3, MAPK8, RB1, TP53* |
| hsa04151 | PI3K-Akt signaling pathway | 30 | 1.40E-28 | *AKT1, FASLG, BAD, CCND1, BCL2, CASP9, CDK4, CDKN1A, CHRM1, CHUK, COL1A1, EGF, EGFR, ERBB2, IKBKB, IL2, IL4, IL6, KDR, MYC, NOS3, PIK3CG, PRKCA, MAPK1, MAPK3, PTEN, RELA, SPP1, TP53, VEGFA* |
| hsa04659 | Th17 cell differentiation | 21 | 2.65E-28 | *AHR, CHUK, MAPK14, FOS, HIF1A, IFNG, IKBKB, IL1B, IL2, IL4, IL6, JUN, NFKBIA, PPP3CA, MAPK1, MAPK3, MAPK8, RELA, STAT1, STAT3, TGFB1* |
| hsa05205 | Proteoglycans in cancer | 25 | 5.88E-28 | *AKT1, FASLG, CCND1, CASP3, CAV1, CDKN1A, MAPK14, EGFR, ERBB2, ESR1, HIF1A, IL6, KDR, MMP2, MMP9, MYC, PLAU, PRKCA, MAPK1, MAPK3, STAT3, TGFB1, TNF, TP53, VEGFA* |
| hsa04010 | MAPK signaling pathway | 28 | 6.24E-28 | *AKT1, FASLG, CASP3, CHUK, MAPK14, EGF, EGFR, ERBB2, FOS, HSPB1, IKBKB, IL1A, IL1B, IL6, JUN, KDR, MYC, PPP3CA, PRKCA, MAPK1, MAPK3, MAPK8, RASA1, RELA, TGFB1, TNF, TP53, VEGFA* |

**SUPPLEMENTARY TABLE 4 |** Key ingredients from XBS identified by UPLC-Q/Orbitrap HRMS analysis.

| **No.** | **Compound name** | **Peak appearance time** | **Molecular Formula** | **Ion detected** | | | **Deviation/**  **ΔPPM/** |
| --- | --- | --- | --- | --- | --- | --- | --- |
|  |  |  |  | **Ion species** | **Theoretical value (m/z)** | **Measured value (m/z)** |  |
| 1 | Liqiritin | 1.64 | C_21_H_22_O_9_ | [M+Na] + | 441.1156 | 441.1167 | 2.6 |
| 2 | Quercetin | 1.98 | C_15_H_10_O^7^ | [M+H] + | 303.0499 | 303.0503 | 1.2 |
| 3 | Kaempferol | 16.47 | C_15_H_10_O_6_ | [M+H] + | 287.0550 | 287.0555 | 1.8 |
| 4 | Licochalcone A | 21.59 | C_21_H_22_O_4_ | [M+H] + | 339.1591 | 339.1063 | 3.7 |
| 5 | Glycyrrhetinic acid | 30.68 | C_30_H_46_O_4_ | [M+H] + | 471.3469 | 471.3480 | 2.4 |

**SUPPLEMENTARY TABLE 5 |** The affinity of ingredients with NF-κB p65/RELA.

| **Chemical** | **Number** | **PDB_ID** | **Total score** | **crash** | **polar** | **C_SCORE** |
| --- | --- | --- | --- | --- | --- | --- |
| Quercetin | 1 | 3NR1 | 11.9543 | -1.4725 | 9.3167 | 3 |
|  | 2 | 5URN | 6.5812 | -1.3918 | 5.6755 | 2 |
|  | 3 | 6QHM | 6.2837 | -1.6604 | 3.7482 | 5 |
|  |  |  |  |  |  |  |
| Kaempferol | 1 | 3NR1 | 7.1296 | -0.9766 | 2.2718 | 2 |
|  | 2 | 5URN | 8.6113 | -1.6672 | 3.436 |  |
|  | 3 | 6QHM | 7.337 | -1.6141 | 4.3835 | 4 |
|  |  |  |  |  |  |  |
| Licochalcone A | 1 | 3NR1 | 7.7152 | -1.8493 | 2.1172 |  |
|  | 2 | 5URN | 6.9684 | -2.1467 | 1.0164 | 2 |
|  | 3 | 6QHM | 7.0063 | -1.805 | 1.0411 | 1 |
|  |  |  |  |  |  |  |
| Liquiritin | 1 | 3NR1 | 12.7512 | -1.958 | 7.6268 | 2 |
|  | 2 | 5URN | 8.6946 | -1.7077 | 5.5982 | 2 |
|  | 3 | 6QHM | 10.716 | -2.5704 | 7.7218 | 4 |

**SUPPLEMENTARY TABLE 6 |** The affinity of ingredients with Akt1.

| **Chemical** | **Number** | | **PDB_ID** | **Total score** | **crash** | **polar** | **C_SCORE** |
| --- | --- | --- | --- | --- | --- | --- | --- |
| Quercetin | 1 | 3D0E | | 7.4194 | -0.8548 | 4.872 |  |
|  | 2 | 6BUU | | 7.6115 | -0.9721 | 5.3624 | 2 |
|  | 3 | 6HHI | | 7.0554 | -1.2588 | 6.3063 | 4 |
|  |  |  | |  |  |  |  |
| Kaempferol | 1 | 3D0E | | 7.8652 | -2.2284 | 3.5437 | 4 |
|  | 2 | 6BUU | | 8.8593 | -1.9134 | 3.8111 | 5 |
|  | 3 | 6HHI | | 10.5769 | -1.6482 | 5.4201 | 2 |

**SUPPLEMENTARY TABLE 7 |** The affinity of ingredients with Caspase-3.

| Chemical | Number | PDB_ID | Total score | crash | polar | C_SCORE |
| --- | --- | --- | --- | --- | --- | --- |
| Quercetin | 1 | 2CNO | 5.2954 | -1.2455 | 4.6233 | 3 |
|  | 2 | 3GJR | 6.2988 | -1.2352 | 4.4812 |  |
|  | 3 | 3KJF | 5.8362 | -1.1883 | 5.3092 | 1 |
|  |  |  |  |  |  |  |
| Kaempferol | 1 | 2CNO | 6.4198 | -1.2104 | 2.3269 | 3 |
|  | 2 | 3GJR | 6.8641 | -0.8995 | 0.9976 | 1 |
|  | 3 | 3KJF | 8.0487 | -2.2418 | 4.532 | 5 |

**SUPPLEMENTARY TABLE 8 |** The affinity of inhibitors with NF-κB p65/RELA, Akt1 and Caspase-3.

| **Chemical** | **Target** | **Number** | **PDB_ID** | **Total score** | **crash** | **polar** | **C_SCORE** |
| --- | --- | --- | --- | --- | --- | --- | --- |
| Bortezomib | NF κB p65 | 1 | 3NR1 | 10.0124 | -1.7144 | 6.4056 | 1 |
|  |  | 2 | 5URN | 8.1419 | -2.6116 | 4.5894 | 0 |
|  |  | 3 | 6QHM | 9.7261 | -1.9325 | 2.392 | 0 |
|  |  |  |  |  |  |  |  |
| Triptolide | NF κB p65 | 1 | 3NR1 | 8.8168 | -1.5576 | 2.9481 | 0 |
|  |  | 2 | 5URN | 7.0776 | -0.4437 | 0.551 | 4 |
|  |  | 3 | 6QHM | 5.0878 | -0.9628 | 0.0162 | 4 |
|  |  |  |  |  |  |  |  |
| MK-2206 2HCl | Akt1 | 1 | 3D0E | 10.5375 | -1.0082 | 4.5691 | 4 |
|  |  | 2 | 6BUU | 10.2994 | -0.8796 | 3.927 | 2 |
|  |  | 3 | 6HHI | 12.9023 | -4.0141 | 4.702 | 0 |
|  |  |  |  |  |  |  |  |
| Z-Devd-fmk | Caspase-3 | 1 | 2CNO | 6.2355 | -2.4427 | 4.9812 | 2 |
|  |  | 2 | 3GJR | 9.3081 | -2.2407 | 4.3341 | 3 |
|  |  | 3 | 3KJF | 9.5683 | -2.9584 | 5.5834 | 4 |
